# Evidence‐based treatment recommendations for neck and low back pain across Europe: A systematic review of guidelines

**DOI:** 10.1002/ejp.1679

**Published:** 2020-11-12

**Authors:** Nadia Corp, Gemma Mansell, Siobhán Stynes, Gwenllian Wynne‐Jones, Lars Morsø, Jonathan C. Hill, Danielle A. van der Windt

**Affiliations:** ^1^ Primary Care Centre Versus Arthritis School of Medicine Keele University Staffordshire UK; ^2^ Department of Psychology School of Life & Health Sciences Aston University Birmingham UK; ^3^ Haywood Hospital Spinal Interface Service Midlands Partnership Foundation NHS Trust Staffordshire UK; ^4^ Open Patient data Explorative Network Department of Clinical Research University of Southern Denmark Odense Denmark

## Abstract

**Background and objective:**

This systematic review synthesized evidence from European neck and low back pain (NLBP) clinical practice guidelines (CPGs) to identify recommended treatment options for use across Europe.

**Databases and Data Treatment:**

Comprehensive searches of thirteen databases were conducted, from 1st January 2013 to 4th May 2020 to identify up‐to‐date evidence‐based European CPGs for primary care management of NLBP, issued by professional bodies/organizations. Data extracted included; aim and target population, methods for development and implementation and treatment recommendations. The AGREE II checklist was used to critically appraise guidelines. Criteria were devised to summarize and synthesize the direction and strength of recommendations across guidelines.

**Results:**

Seventeen CPGs (11 low back; 5 neck; 1 both) from eight European countries were identified, of which seven were high quality. For *neck pain*, there were consistent *weak* or *moderate* strength recommendations for: reassurance, advice and education, manual therapy, referral for exercise therapy/programme, oral analgesics and topical medications, plus psychological therapies or multidisciplinary treatment for specific subgroups. Notable recommendation differences between back and neck pain included, i) analgesics for neck pain (not for back pain); ii) options for back pain‐specific subgroups—work‐based interventions, return to work advice/programmes and surgical interventions (but not for neck pain) and iii) a greater strength of recommendations (generally moderate or strong) for back pain than those for neck pain.

**Conclusions:**

This review of European CPGs identified a range of mainly non‐pharmacological recommended treatment options for NLBP that have broad consensus for use across Europe.

**Significance:**

Consensus regarding evidence‐based treatment recommendations for patients with neck and low back pain (NLBP) from recent European clinical practice guidelines identifies a wide range of predominantly non‐pharmacological treatment options. This includes options potentially applicable to all patients with NLBP and those applicable to only specific patient subgroups. Future work within our Back‐UP research team will transfer these evidence‐based treatment options to an accessible clinician decision support tool for first contact clinicians.

## 1 INTRODUCTION

Neck and low back pain (NLBP) are among the most frequent reasons for visiting a general practitioner (GP) or physiotherapist in primary care in Europe (Bot et al., 2005; Jordan et al., 2010). The substantial burden of illness from these conditions was shown by the most recent Lancet‐Global Burden of Disease study which highlighted low back pain (LBP) as the single highest cause of years lived with disability (out of 354 conditions studied), with neck pain ranked eighth (female) and twelfth (male) (GBD 2017  Disease and Injury Incidence and Prevalence Collaborators, 2018). Outlining potential ways to address this societal burden, the recent Lancet series on LBP (Foster et al., 2018) recommended a greater focus on improving decision making in first‐contact consultations as current treatment is highly variable (Maserejian et al., 2014) and often not in line with clinical guidelines (Darlow et al., 2014; Somerville et al., 2008), leading to suboptimal treatment outcomes (Maher et al., 2017). For example referrals to secondary care specialists are too common, provision of self‐management advice and education can be limited, opioids and imaging are over‐prescribed, and sign‐posting to locally available non‐pharmacological options such as exercise groups are limited (Chou, et al., 2017a; Koes et al., 2010; Maserejian et al., 2014). Finding solutions that promote best‐practice care for patients with NLBP in first‐contact consultations is therefore a priority (Foster et al., 2012).

Our team is part of Back‐UP, a European programme of research developing a digital health technology to support clinical decision making for patients with NLBP based on a stratified care approach for first‐contact consultations [http://backup‐project.eu/]. Decision support tools have demonstrated promising results for helping clinicians to translate the most up to date recommended evidence into their practice (Murphy et al., 2014). For example a systematic review of over 160 randomized controlled trials testing clinical decision‐support systems identified improved processes of clinical care (e.g. diagnosis, treatment, disease monitoring) or patient outcomes (e.g. clinical events, quality of life) in over half of the included studies (Roshanov et al., 2013).

The Keele STarT Back stratified care Tool for back pain has recently been superseded by the Keele STarT MSK Tool (Dunn et al., submited), which has been validated in UK primary care and shown to be predictive of pain and disability across a range of common musculoskeletal (MSK) pain sites, including NLBP. In addition, a new set of recommended matched treatment options for MSK patients at low, medium and high‐risk of poor outcomes (Babatunde et al., 2017; Protheroe et al., 2019) have been piloted in a feasibility trial (Hill et al., 2020). However, these matched treatments were designed to evaluate stratified care in UK general practice rather than for use across European countries by a broader range of first‐contact clinicians such as occupational health physicians. We therefore felt the matched treatments should be further refined for the specific context of this European project.

Recent systematic reviews of clinical practice guidelines (CPGs) for musculoskeletal pain (Lin et al., 2020), and back pain (Oliveira et al., 2018; Wong et al., 2017) aimed to summarize recommended treatments for either LBP or neck pain. However, less emphasis was placed on improving decision making in first‐contact consultations, identifying specific CPG recommendations for patient subgroups defined by their risk of persistent pain and disability and the potential relevance, and on improving the referral process. To our knowledge, no prior reviews of CPGs have assessed treatment recommendations for both neck and low back pain and explored consistencies or similarities between recommendations for these common spinal pain presentations.

The aim of this study was therefore to conduct a systematic review of published European back and neck pain clinical guidelines to describe and synthesize the evidence of recommended treatment options with broad consensus for use across Europe.

## 2 METHODS

A systematic review of contemporary European clinical practice guidelines was conducted and reported following the Preferred Reporting Items for Systematic Reviews and Meta‐Analyses guidance (PRISMA; Moher et al., 2009).

### 2.1 Systematic review protocol

An a priori protocol was written and followed (Available at http://backup‐project.eu/?page_id=84).

### 2.2 Search strategy

A comprehensive search strategy was conducted of eight electronic databases (EMBASE, MEDLINE, CINAHLPlus, HMIC, PsycINFO, Epistemonikos, Pedro and TRIP database) and five sources of grey literature (National Institute for Health and Clinical Excellence (NICE), Scottish Intercollegiate Guidelines (SIGN), WHO Guidelines, Guidelines International Network (G‐I‐N) and DynaMed Plus) from 1st January 2013 to 4th May 2020.

The search strategy utilized both text word searching in the title, abstract or keywords and database subject headings, combining terms for neck or back pain and practice guidelines (see Appendix S1: full‐search strategy for OVID MEDLINE). For the other databases, search terms were adapted to the search capabilities of the platform.

In addition, our Back UP research partners were asked to identify any relevant guidelines from their country. Reference lists of included guidelines were checked to identify additional documents relevant to the methodology of the guideline.

### 2.3 Eligibility criteria

#### 2.3.1 Inclusion criteria

- Recent evidence‐based European clinical guidelines issued by professional bodies or organizations for guideline development [published from 2013 onwards]. We included recently published guidance only, to ensure treatment recommendations emerging from the review would be based on relatively up‐to‐date evidence;
- Guidelines concern adult populations (18 years or over), with NLBP (including patients presenting to first contact health professionals with symptoms of whiplash‐related disorders or symptoms of radiculopathy such as radicular pain);
- Guidelines that include recommendations regarding treatment options for patients presenting with NLBP, in particular:
  - Treatments deliverable within primary care (as broadly considered across Europe, including occupational healthcare) or referral pathways from primary to secondary care recommended for clinical practice (in at least two European countries).
  - Treatments aiming to reduce pain, improve function and/or support return to work. Relevant outcomes also included evidence‐based recommendations regarding factors (patient, clinician, environment) that may be associated with the effectiveness of treatment, and recommendations regarding clinical prediction rules or decision tools supporting the selection of treatment for specific patient subgroups (where mentioned in the guideline).

#### 2.3.2 Exclusion criteria

- Non‐European guidelines;
- All publications that are not evidence‐based clinical practice guidelines, including guidelines based solely on consensus or without an explicit literature search, and other publication types: systematic reviews, randomized trials, cohort studies, case series, editorials, protocols, letters;
- Paediatric only populations (under 18 years);
- NLBP as a result of severe trauma, for example fracture and spinal cord injury, inflammatory arthritis including spondyloarthropathies, and those that focused on broader conditions, for example (chronic) pain that may encompass spinal pain;
- Guidelines focused on patients managed in secondary care with an established diagnosis of radiculopathy;
- Guidelines focused specifically on surgical treatment options/comparisons or on specific interventions not limited to spinal pain, for example analgesics in older adults;
- Guidelines that involved populations admitted to hospital (not ambulatory care);
- Guidelines for which translations could not be obtained.

### 2.4 Guideline selection

Results from all searches were imported into EndNote X9 (reference management software, Clarivate Analytics. Available at https://endnote.com/) and duplicates removed. Unique citations were then imported into Covidence (Veritas Health Innovation, Melbourne, Australia. Available at https://www.covidence.org/) to manage the screening process.

Two reviewers (NC and GM) independently screened all titles and abstracts for relevance against eligibility criteria and excluded ineligible publications by agreement. Full texts were independently assessed for inclusion by pairs of independent reviewers (NC, GM and DvdW). Disagreements were noted and resolved between pairs of reviewers and where necessary the involvement of a third reviewer. Reasons for exclusion at the full‐text stage were recorded.

### 2.5 Data extraction

A data extraction form was purposively designed in Excel to record relevant information from each of the clinical practice guidelines included in the review. Complementary documents were sourced where relevant, such as methodological reports and evidence syntheses. Information was extracted regarding general guideline information (e.g. country, healthcare setting, publication year, target population and presenting symptoms); methods regarding guideline development and implementation (e.g. multidisciplinary group/single profession; how strength of evidence determined; details regarding consensus methods) and intervention recommendations, specifically only those that can be offered in primary care, and guidance for referral (e.g. [strength of] recommendations, any details regarding subgroups).

One reviewer extracted data from each guideline; in the case of guidelines in English, this was independently checked by a second reviewer with any disagreements resolved through discussion. For non‐English guidelines, no independent check with a second experienced reviewer was feasible within the timeline of conducting this review.

### 2.6 Assessment of guideline quality

The AGREE II (Appraisal of Guidelines Research and Evaluation) reporting checklist was used to critically appraise guidelines (Brouwers et al., 2010a). Internationally, this is the most widely used tool for assessing guideline quality (Siering et al., 2013), with good construct validity and reliability (Brouwers et al., 2010b, 2010c). The instrument focuses on guideline development and reporting and consists of 23 items addressing 6 domains (1. Scope and purpose; 2. Stakeholder involvement; 3. Rigour of development; 4. Clarity of presentation; 5. Applicability and 6. Editorial independence). Each item is rated on a 7‐point Likert scale from 1 (Strongly disagree) to 7 (Strongly agree). In addition, there are two final items that ask appraisers to give an overall judgement in light of ratings given for the 23 items.

The web‐based platform My AGREE PLUS (https://www.agreetrust.org/my‐agree/) was used to complete appraisals online, based on the user manual. Each item is presented for scoring alongside detailed guidance on how to score the item, including where to find relevant information and what to consider when deciding on the score for each item.

Critical appraisal was conducted concurrent to data extraction by the same reviewer(s). One reviewer appraised each guideline; in the case of guidelines in English, this was independently checked by a second reviewer with any disagreements resolved through discussion. For non‐English guidelines no independent check was feasible.

No set thresholds exist for determining high‐/low‐quality guidelines, however, AGREE II guidance suggests users decide these according to their specific context (AGREE Next Steps Consortium, 2017). On the basis of the examples given in the AGREE II user manual, and with reference to previous studies (Bouwmeester et al., 2009; Lin et al., 2020), we considered guidelines to be of high quality if AGREE II Domain 3, that is ‘Rigour of development’ scored at least 70%, and the remaining five domains, along with the overall assessment, scored at least 50%.

### 2.7 Synthesis of guidelines and identification of consistent recommendations

All recommendations extracted from the included guidelines were collated based on the way the treatment option was described in/translated from the guideline and then grouped according to treatment theme. Tables were drawn up to present all the recommendations, alongside details regarding the context of the guideline (i.e. professional organization(s), country and target population/diagnostic classification). Members of the review team, which included researchers with academic and clinical expertise in musculoskeletal pain, were presented with these tables for review. Following discussion of the many very specific intervention options, for example different forms of exercise, nuanced and/or inconsistently used terms and translation anomalies/country‐specific terminology (often reported in only 1 or 2 guidelines), interventions were merged by treatment type/modality. A general practitioner (physician) was invited to review the recommendations relating to medications specifically and undertook a similar process of refining the grouping of treatment options.

The direction (*i.e*. for, against or open) and strength of recommendations were harmonized, taking into consideration the array of methods and terminologies used across included guidelines (see Appendix S2). The resulting nomenclature enables the reader to distinguish between strong or weak recommendations based on a formal grading system, for example GRADE; those where no formal grading system was applied and recommendations based on consensus/expert opinion. Treatment or referral options for which a recommendation was formulated in one guideline only, were not further considered.

To summarize and synthesize the direction and strength of recommendations across guidelines, a set of criteria was devised and followed, such that:

- **Strong FOR/AGAINST recommendation** (should do/should not do): consistent recommendations in at least two high‐quality guidelines from different countries (at least one guideline of which reports a 'strong' *i.e*.// or XX recommendation).
- **Moderate FOR/AGAINST recommendation** (could do/could not do): consistent recommendations in at least one high quality (where the recommendation is not based on expert opinion *i.e*. O + or O‐) and if only one high quality, then one or more low‐quality guidelines.
- **Weak FOR/AGAINST recommendation:** recommendations from high‐quality guidelines but based on expert opinion only and/or recommendations from multiple low‐quality guidelines.
- **Inconsistent**: inconsistent recommendations from guidelines of high quality (for/against).
- **Inconclusive**: open/uncertain recommendations only, or recommendations from low‐quality guidelines are inconsistent.

## 3 RESULTS

### 3.1 Guideline selection

**Figure 1:**
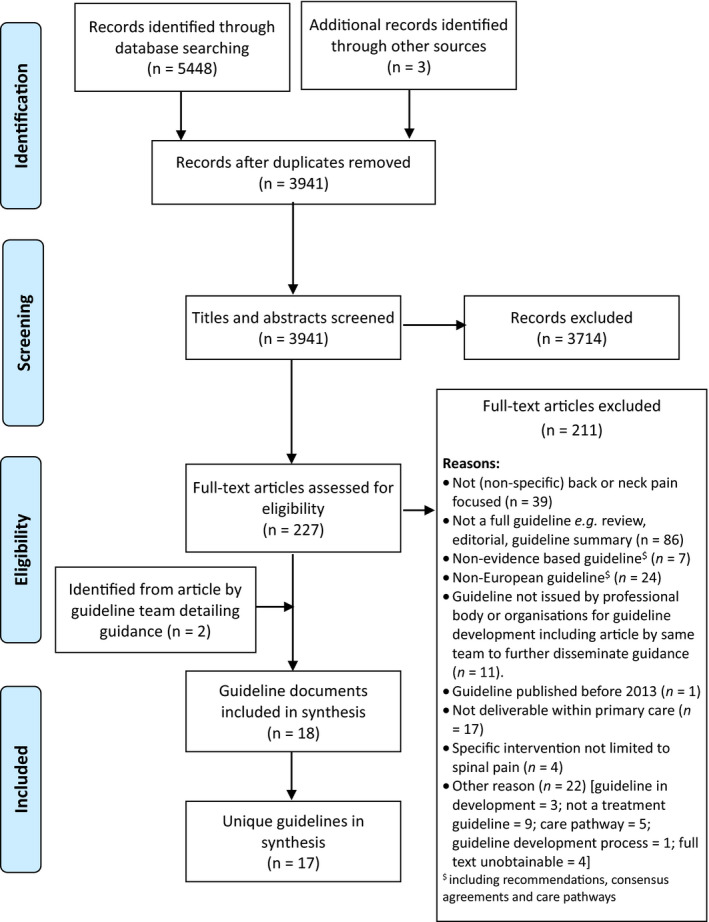
PRISMA Flow Diagram

The systematic search resulted in 3,941 unique citations, from which 17 clinical practice guidelines (CPGs) were identified (Figure 1) and included in this evidence synthesis (Bier et al., 2016; Bons et al., 2017; BÄK et al., 2017; Glocker et al., 2018; Kassolik et al., 2017; Monticone et al., 2013; NICE, 2016; Pohl et al., 2018; Regione Toscana, 2015; Schaafstra et al., 2015; SFMT, 2013; Staal et al., 2017; Sundhedsstyrelsen, 2015; 2016a, 2016b, 2016c; van Wambeke et al., 2017).

### 3.2 Guideline characteristics

An overview of characteristics of included CPGs and the methods used in their development and implementation is presented in Table 1, with guideline‐specific details provided in Appendices S3 & S4. The 17 contemporary CPGs originate from eight European countries (Belgium, Denmark, France, Germany, Italy, The Netherlands, Poland and the UK). The majority address low back pain and/or radicular pain (*n* = 12; 71%), whereas six (35%) are concerned with neck pain and/or radicular pain. Five guidelines (29%) focus specifically on patients presenting with symptoms of radiculopathy. Three of these guidelines (Schaafstra et al., 2015; Sundhedsstyrelsen, 2015; 2016b) are specifically developed for the management of radiculopathy in general practice or primary care. The two other guidelines were designed for healthcare professionals responsible for the management of acute lumbar (Glocker et al., 2018) or cervical (Pohl et al., 2018) radiculopathy in any ambulant, outpatient or secondary care setting. Conversely, three CPGs (18%) explicitly exclude radiculopathy.

**Table 1:** Characteristics of included clinical practice guidelines

**Characteristic**	***n***	**Reference**
**Country**		
Belgium	1	van Wambeke et al., 2017
Denmark	4	Sundhedsstyrelsen, 2015, 2016a,2016b,2016c
France	1	SFMT, 2013
Germany	3	BÄK et al., 2017; Glocker et al., 2018; Pohl et al., 2018
Italy	2	Monticone et al., 2013; Regione Toscana, 2015
The Netherlands	4	Schaafstra et al., 2015; Bier et al., 2016; Bons et al., 2017; Staal et al., 2017
Poland	1	Kassolik et al., 2017
UK	1	NICE, 2016
**Pain site**		
Neck	5	Monticone et al., 2013; Sundhedsstyrelsen, 2015; Bier et al., 2016; Sundhedsstyrelsen, 2016c; Pohl et al., 2018
Low back	11	SFMT, 2013; Regione Toscana, 2015; Schaafstra et al., 2015; NICE, 2016; Sundhedsstyrelsen, 2016a,2016b; BÄK et al., 2017; Bons et al., 2017; Staal et al., 2017; van Wambeke et al., 2017; Glocker et al., 2018
Neck & low back	1	Kassolik et al., 2017
**Specifically excludes radiculopathy**		
Neck	1	Sundhedsstyrelsen, 2016c
Low back	3	Sundhedsstyrelsen, 2016a; BÄK, et al., 2017; Bons et al., 2017
**Radiculopathy only focus**		
Neck	2	Sundhedsstyrelsen, 2015; Pohl et al., 2018
Low back	3	Schaafstra et al., 2015; Sundhedsstyrelsen, 2016b; Glocker et al., 2018
**Multidisciplinary group or single profession**		
Multidisciplinary	14	SFMT, 2013; Regione Toscana, 2015; Schaafstra et al., 2015; Sundhedsstyrelsen, 2015; NICE, 2016; Sundhedsstyrelsen, 2016a, 2016b, 2016c; BÄK et al., 2017; Bons et al., 2017; Kassolik et al., 2017; van Wambeke et al., 2017; Glocker et al., 2018; Pohl et al., 2018
Single	2	Bier et al., 2016; Staal et al., 2017
Not reported	1	Monticone et al., 2013
**Formal grading of evidence and/or recommendation**		
Yes	13	Monticone et al., 2013; SFMT, 2013; Regione Toscana, 2015; Sundhedsstyrelsen, 2015, Bier et al., 2016; NICE, 2016; Sundhedsstyrelsen, 2016a, 2016b, 2016c; BÄK et al., 2017; Kassolik et al., 2017; Staal et al., 2017; van Wambeke et al., 2017; Pohl et al., 2018
No	3	Schaafstra et al., 2015; Bons et al., 2017; Glocker et al., 2018
Not reported	1	Kassolik et al., 2017
**Details of consensus process given**		
Yes	8	SFMT, 2013; Schaafstra et al., 2015; NICE, 2016; BÄK et al., 2017; Bons et al., 2017; Glocker et al., 2018; Pohl et al., 2018; van Wambeke et al., 2017
No	9	Monticone et al., 2013; Regione Toscana, 2015; Sundhedsstyrelsen, 2015; Bier et al., 2016; Sundhedsstyrelsen, 2016a, 2016b, 2016c; Kassolik et al., 2017; Staal et al., 2017
**Includes recommendations regarding**		
Future revision	10	Sundhedsstyrelsen, 2015; NICE, 2016; Sundhedsstyrelsen, 2016a, 2016b, 2016c; BÄK, et al., 2017; Staal et al., 2017; van Wambeke et al., 2017; Glocker et al., 2018; Pohl et al., 2018
Evaluation of red flags	12	Monticone et al., 2013; SFMT, 2013; Regione Toscana, 2015; Schaafstra et al., 2015; Bier et al., 2016; NICE, 2016; BÄK et al., 2017; Staal et al., 2017; van Wambeke et al., 2017; Bons et al., 2017; Glocker et al., 2018; Pohl et al., 2018
Evaluation of yellow flags	10	SFMT, 2013; Regione Toscana, 2015; Bier et al., 2016; NICE, 2016; BÄK et al., 2017; Bons et al., 2017; Staal et al., 2017; van Wambeke et al., 2017; Pohl et al., 2018; Glocker et al., 2018
Evaluation of blue/black flags	7	SFMT, 2013; Regione Toscana, 2015; Bier et al., 2016; BÄK, et al., 2017; Bons et al., 2017; Staal et al., 2017; van Wambeke et al., 2017
Diagnosis	12	Monticone et al., 2013; SFMT, 2013; Regione Toscana, 2015; Schaafstra et al., 2015; Bier et al., 2016; NICE, 2016; BÄK et al., 2017; Bons et al., 2017; Kassolik et al., 2017; Staal et al., 2017; Pohl et al., 2018; Glocker et al., 2018
Planning of care	14	Monticone et al., 2013; Bier et al., 2016; Pohl et al., 2018; Sundhedsstyrelsen, 2015, 2016a, 2016b, 2016c; BÄK et al., 2017; Bons et al., 2017; NICE, 2016; Regione Toscana, 2015; Schaafstra et al., 2015; Staal et al., 2017; van Wambeke et al., 2017, ^a^
Practitioner education	8	Sundhedsstyrelsen, 2015; Regione Toscana, 2015; Sundhedsstyrelsen, 2016a, 2016b, 2016c; NICE, 2016; van Wambeke et al., 2017, ^a^; Pohl et al., 2018
Organization & policy	5	SFMT, 2013; Regione Toscana, 2015; BÄK et al., 2017; Bons et al., 2017; van Wambeke et al., 2017, ^a^

^a^ subsequent clinical pathway developed that addressed this issue (Jonckheer et al., 2017)

A large majority of CPGs were developed by multidisciplinary groups (*n* = 14, 82%), employed formal grading of evidence and/or recommendations (*n* = 13, 76%). Just over half the guidelines detailed timeframes for future revisions (*n* = 10, 59%), whereas just under half detailed or undertook a consensus process (*n* = 8, 47%).

In addition to treatment recommendation most guidelines addressed planning of care (*n* = 14, 82%), diagnostic assessment (*n* = 12, 71%), evaluation of red (*n* = 12, 71%) and/or yellow (psychosocial, *n* = 10, 59%) flags. Conversely, less than half the guidelines detailed the evaluation of blue/black flags, that is blue: individuals’ perceptions of work‐related factors and the relationship between work and health, black: system‐level factors (context, work environment, policies) (*n* = 7, 41%), practitioner education (*n* = 8, 47%) or organization and policy implications (*n* = 5, 29%).

### 3.3 Quality appraisal

The AGREE II domain scores for each guideline are presented in Table 2, along with our designation of the overall quality, that is high/low based on domain scores. Notably, one guideline (Kassolik et al., 2017) was not rated highly on any of the domains, achieving at its best 44% for clarity of presentation. With the exception of this guideline, the remaining 16/17 CPGs were all highly rated, achieved at least 50% of the maximum possible score, for Domains 1 (scope and purpose) and 4 (clarity of presentation). Conversely, a minority of CPGs (*n* = 7, 41%) achieved high ratings for Domain 5 (applicability). Domains 2 (stakeholder involvement) and 6 (Editorial independence), together with overall assessment score, were each reported to a high quality in a large majority of studies (*n* = 14, 82%). Domain 3 (rigour of development) with its higher cut‐off point of 70% determining high quality was achieved by just over half the CPGs (*n* = 9, 53%).

**Table 2:** Quality appraisal of guidelines: AGREE II domain scores (%) and quality assessment. Cells in green indicate domain attained ‘high’ rating.

Guideline	Domain 1. Scope and Purpose	Domain 2. Stakeholder Involvement	Domain 3. Rigour of Development	Domain 4. Clarity of Presentation	Domain 5. Applicability	Domain 6. Editorial In‐dependence	Overall quality of guideline	Guideline recommended for use	Quality (high/low)
Neck pain only									
Bier et al., 2016	**72%**	**94%**	52%	**67%**	38%	**50%**	**67%**	Yes	**Low**
Monticone et al., 2013	**50%**	33%	38%	**100%**	0%	**50%**	33%	Yes, with modifications^a^	**Low**
Pohl et al., 2018	**61%**	**72%**	54%	**89%**	17%	**92%**	**50%**	No	**Low**
Sundhedsstyrelsen, 2015	**78%**	**67%**	**71%**	**72%**	**58%**	**83%**	**67%**	Yes, with modifications^a^	**High**
Sundhedsstyrelsen, 2016c	**89%**	**72%**	**75%**	**67%**	**63%**	**83%**	**83%**	Yes	**High**
Back pain only									
BÄK et al., 2017	**89%**	**89%**	**77%**	**94%**	**79%**	**75%**	**83%**	Yes	**High**
Bons et al., 2017	**72%**	**78%**	**81%**	**61%**	29%	**83%**	**83%**	Yes	**Low**
Glocker et al., 2018	**72%**	44%	44%	**56%**	21%	**100%**	33%	No	**Low**
NICE, 2016	**100%**	**78%**	**79%**	**94%**	**79%**	**58%**	**83%**	Yes	**High**
Regione Toscana, 2015	**83%**	**78%**	48%	**100%**	25%	17%	**50%**	Yes, with modifications^a^	**Low**
Schaafstra et al., 2015	**72%**	**78%**	65%	**50%**	29%	**83%**	**50%**	Yes with modifications^a^	**Low**
SFMT, 2013	**89%**	**72%**	65%	**100%**	13%	**100%**	**83%**	Yes	**Low**
Staal B. et al., 2017	**83%**	**83%**	**77%**	**94%**	33%	42%	**67%**	Yes with modifications^a^	**Low**
Sundhedsstyrelsen, 2016a	**78%**	**67%**	**71%**	**61%**	**63%**	**83%**	**67%**	Yes, with modifications^a^	**High**
Sundhedsstyrelsen, 2016b	**89%**	**72%**	**79%**	**72%**	**63%**	**83%**	**83%**	Yes	**High**
van Wambeke et al., 2017	**100%**	**83%**	**92%**	**94%**	**71%**	**100%**	**100%**	Yes	**High**
Neck and back pain									
Kassolik et al., 2017	39%	22%	4%	44%	8%	42%	33%	No	**Low**

^a^ AGREE II user manual provides no guidance on what this actually means and so is open to different interpretations by the different reviewers. But, broadly this was taken to mean a guideline was close to being recommended for use, but just need a little more detail in one or two areas.

Seven CPGs (41%) were considered high‐quality overall: 2 focused on neck pain, both Danish (Sundhedsstyrelsen, 2015; 2016c) and 5 on low back pain including 1 Belgian, 1 UK, 2 Danish and 1 German (BÄK et al., 2017; NICE, 2016; Sundhedsstyrelsen, 2016a; 2016b; van Wambeke et al., 2017), (Table 2 and Appendices S5 & S6).

### 3.4 Consistency of CPG recommendations for neck pain

Six guidelines provided treatment recommendations for neck pain (Bier et al., 2016; Kassolik et al., 2017; Monticone et al., 2013; Pohl et al., 2018; Sundhedsstyrelsen, 2015; 2016c). Appendix S5 details the specific treatment options or intervention modalities identified in each guideline together with the direction and strength of each recommendation. In total, recommendations were provided that covered a wide range of treatment options: reassurance; advice and education; medication; injection/infiltration; acupuncture; thermotherapy; manual therapy; exercise therapy; postural therapy; traction; electrotherapy; orthotics; ergonomic interventions; taping/strapping; psychological interventions; multidisciplinary treatments; referral for imaging and referral for specialist opinion; plus a disparate group of interventions that were labelled ‘miscellaneous’.

In considering the consistency of recommendations across all neck pain CPGs (Table 3), 14 treatment options were supported, whereas recommendations were inconsistent or inconclusive (mixed) regarding the use of seven treatment options. For 26 treatment options, a recommendation was only given in one guideline, and these were not further considered.

**Table 3:** Consistency of recommendations across guidelines for neck pain (see Appendix S5 for individual guidelines). *Symbol – classification:* // ‐ should do; / ‐ could do; /* ‐ for (generic); O [O+/O‐] – Open [expert opinion in favour/against]; X* ‐ against (generic); X – should not do; XX – definitely do not do (see Supporting Information Appendix S2 for further detail).

Intervention	No. guidelines (countries)	Recommendations by guideline quality		Overall strength of recommendation	Comments
		HIGH quality	LOW quality		
FOR					
Reassurance	3(3)	1x **O+**	1x **/**; 1x /*	Weak FOR	
Advice and Education	5(5)	1x **O+**	**For:** 1x **//**; 1x **/;** 1x /* **Against:** 1x **X**	Weak FOR	
Remain active (advice)	2(2)	1x **O+**	1x **/**	Weak FOR	
Encourage exercise (advice)	3(3)	1x **O+**	1x **/**; 1x /*	Weak FOR	
Analgesics incl. for neuropathic pain	2(2)		1x **//**; 1x /*	Weak FOR	
Paracetamol	2(2)	1x **O+**	1x **/**	Weak FOR	
NSAIDs	4(3)	2x **O+**	1x **/**; 1x /*	Weak FOR	Short‐term use
Opioids including tramadol	2(1)	2x **O+**		Weak FOR	Short‐term use
Topical medications incl. NSAIDs	2(2)	1x **/**	1x /*	Moderate FOR	
Manual therapy + other treatment	3(3)	1x **/**	1x **//** & **/**; 1x **/** & **O+**	Moderate FOR	
Exercise programs/therapy	5(5)	1x **/** & **O+**	2x **//**; 1x **/**; 1x /*	Moderate FOR	
Exercise therapy + other treatment	2(2)	1x **/**	1x **//**	Moderate FOR	
Psychological therapies	3(3)		1x **/**; 1x /*; 1x **O+**	Weak FOR SPECIFIC SUBGROUPS	For specific cases: mood problems, psychosocial risks, or complex, persistent pain problems
Multidisciplinary treatment	2(2)		2x **/**	Weak FOR SPECIFIC SUBGROUPS	For those with more complex or persistent pain
MIXED *i.e.* inconsistent or inconclusive					
Thermotherapy	2(2)		**For:** 1x /* **Against:** 1x X*	Inconclusive	
Manual therapies	5(4)	**Mixed:** 1x **/** & **O‐** **Against:** 1x X	**For:** 2x **//**; 1x /*	Inconsistent	
Traction	3(3)	**For:** 1x **/**	**Mixed:** 1x O+ & X* **Against:** 1x **X**	Inconclusive	For specific cases: radiculopathy (Sundhedsstyrelsen, 2015), Grade III, profile D (Bier *et al.,* 2016)
Electrotherapies	4(4)		**Mixed:** 1x **/** & **O+** & **X**; 1 x /* & X* **Against:** 1x **XX**; 1x X*	Inconclusive	
Cervical orthoses	4(4)		**For:** 1x /* **Mixed:** 1x **O+** & X* **Against:** 2x **O‐**	Inconclusive	For specific cases: Grade III, profile D (Bier *et al.,*2016), or short‐term in cases of severe pain (Pohl *et al.,* 2018)
Acupuncture/dry needling	4(3)	**For:** 1x **/** **Against:** 1x **O‐**	**For:** 1x **//** **Against:** 1x X*	Inconsistent	
Imaging	2(2)		**For:** 1x **//** **Against** 1x X*	Inconclusive	
Single guideline recommendation ‐ in favour of: O+, /*, / or //					
Avoid movement that provokes radiating pain or other symptoms in the arm (advice)		Electrotherapies + active treatment		Encourage patient to contact GP, psychologist or psychosomatic therapist	
Psychosocial aspects that delay recovery (advice)		Kinesiology tape		Workplace interventions	
Continue/return to work (advice)		Cervical cushion/pillow		Referral to GP and/or occupational health officer	
Work‐related/occupational advice		Bioptron lamps		Referral to GP or referring specialist	
Thermotherapy + other treatment		Ledotherapy lamps		Referral to physical therapist specialized in worker rehabilitation	
Steroids		Infra‐red lamps		Referral to occupational health and safety service	
Spinal epidural steroid injection (transforaminal route with imaging)		Bath salts with mud extracts, special water‐pearling inserts or ozone		Referral to occupational health officer or a physical therapist specialised in worker rehabilitation	
Postural re‐education		Magnetic mattress		Referral to surgeon/surgery	
Single guideline recommendation ‐ against: O‐, X*, X or XX					
Bed rest (advice) [1‐2 days, selected cases only]		Written information (advice)			

Positive (weak to moderate) recommendations from high quality (Sundhedsstyrelsen, 2015; 2016c) or multiple low quality (Bier et al., 2016; Kassolik et al., 2017; Monticone et al., 2013; Pohl et al., 2018) guidelines supported the use of reassurance; advice and education with the specific mention of physical activity and exercise; prescription of oral analgesic medications including for neuropathic pain, and specifically paracetamol, NSAIDs and opioids including tramadol; topical medication; exercise interventions alone or in combination with other treatments and manual therapy in combination with another (exercise) intervention.

Psychological or multimodal (multidisciplinary) interventions were recommended for specific subgroups of patients with neck pain, with either psychosocial risk factors or for those with more persistent neck pain or disability.

Recommendations were inconsistent or inconclusive regarding manual therapies (delivered without additional active treatment); traction; electrotherapies; thermotherapies; cervical orthoses; acupuncture/dry needling and referral for imaging.

### 3.5 Consistency of CPG recommendations for low back pain

Twelve guidelines provided treatment recommendations for back pain (Bons et al., 2017; BÄK et al., 2017; Glocker et al., 2018; Kassolik et al., 2017; NICE, 2016; Regione Toscana, 2015; Schaafstra et al., 2015; SFMT, 2013; Staal et al., 2017; Sundhedsstyrelsen, 2016a, 2016b; van Wambeke et al., 2017). Details regarding the specific treatment options or intervention modalities identified from each guideline can be found in Appendix S6, along with the direction and strength of each recommendation. Similar to guidelines for neck pain, recommendations were provided that covered a wide range of treatment and referral options. For many of these treatment options, the body of evidence underpinning recommendations was larger compared to neck pain, although often still inconsistent or of low quality.

Table 4 presents the summary of recommendations from high‐ and low‐quality guidelines and the overall recommendations derived from our synthesis. A range of treatment options (*n* = 26) were only mentioned in one guideline, and these were not considered further. Positive (weak to strong) recommendations from high quality (BÄK et al., 2017; NICE, 2016; Sundhedsstyrelsen, 2016a; 2016b; van Wambeke et al., 2017) or multiple low quality (Bons et al., 2017; Glocker et al., 2018; Kassolik et al., 2017; Regione Toscana, 2015; Schaafstra et al., 2015; SFMT, 2013; Staal et al., 2017) guidelines supported the use of 14 treatment options, including the following: reassurance; advice and education with specifics for physical activity, exercises and work; manual therapy in combination with active treatment; exercise interventions; group exercise programmes including back schools; psychological therapies including cognitive behavioural interventions as standalone interventions or in combination with exercise; work‐based rehabilitation and return to work programmes.

**Table 4:** Consistency of recommendations across guidelines for low back pain (see Appendix S6 for individual guidelines). *Symbol – classification:* // ‐ should do; / ‐ could do; /* ‐ for (generic); O [O+/O‐] – Open [expert opinion in favour/against]; X* ‐ against (generic); X – should not do; XX – definitely do not do (see Supporting Information Appendix S2 for further detail)

Intervention	No. guidelines (countries)	Recommendations by guideline quality		Overall strength of recommendation	Comments
		HIGH quality	LOW quality		
**FOR**					
Reassurance	4(4)	1x **O+**	1x **//**; 2x /*	Weak FOR	
Advice and Education (including individualised)	10(8)	1x **//**; 1x **/**; 2x **O+**	**For**: 1x **//**; 4x /* **Mixed**: 1x **O+** & **O**	Strong FOR	
Remain active	9(6)	1x **//** & **O+;** 2x **/**; 2x **O+**	1x **//** & **O+;** 3x /*	Strong FOR	
Encourage physical exercise (unsupervised)	7(6)	2x **O+**	1x **//**; 1x **//** & **O+**; 3x /*	Weak FOR	
Continue/return to work	2(2)		1x **//** & **O+**; 1x /*	Weak FOR	
Manual therapy in combination with other treatment	4(3)	2x **/**; 1x **O+**	1x /*	Moderate FOR	
Exercise programs/therapy	9(6)	**For**: 3x **/** **Mixed**: 1x **//** & **O**	**For:** 4x /* **Against:** 1x **XX**	Strong FOR	
Group exercise programmes/back schools	3(3)	1x **/**; 1x **/** & **O+**	1x /*	Moderate FOR	
Psychological therapies including behavioural and CBT	4(3)	1x **//**	3x /*	Strong FOR SPECIFIC SUBGROUPS	For specific cases: mood problems, psychosocial risks, or complex, persistent pain problems
Psychological therapies in combination with other treatment (exercise)	2(2)	2x /		Moderate FOR	
Multidisciplinary treatment including MBR programs, and multidisciplinary rehabilitation involving work focus	7(5)	1x **//**; 2x **/**	**For**: 2x /*; 1x **O+** **Mixed:** 1x **/** & **O**	Strong FOR SPECIFIC SUBGROUPS	For specific cases: subacute and chronic LBP with patient strongly motivated to resolve and/or psychosocial obstacles to recovery.
Work‐based interventions including rehabilitation programmes	3(3)	1x **/**	1x **//**; 1x **//** & **/**	Moderate FOR	
Return to work programmes	3(3)	1x **//**; 2x **O+**		Strong FOR	
To surgeon/surgery	8(6)	**For**: 1x **//**; 1x **/;** 1x **O+** **Against:** 1x **XX**	**For**: 2x /* **Against**: 1x X* **Mixed:** 1x **O+** & **O**	Strong FOR SPECIFIC SUBGROUPS	For specific cases: failure of non‐surgical treatment, moderate/severe persistent pain; specific indications e.g. cauda equine, severe neurological symptoms etc.
**AGAINST**					
Bed rest	6(4)	1x **XX**	1x **XX**; 4x X*	Strong AGAINST WITH EXCEPTIONS	Except: for a few days in severe/acute cases
Paracetamol	8(6)	**Against:** 3x **X,** 1x X*	**For:** 1x **//** & **O+** & **O**; 3x /*	Moderate AGAINST	
Antidepressants including SSRIs, SNRIs, Tricyclics	6(5)	**Against:** 1x X*; 1x **XX** & **X** **Mixed:** 1x **O+** & **X**	**Against:** 1x X* **Mixed:** 1x /* & X* **Open:** 1x **O**	Strong AGAINST WITH EXCEPTIONS	For specific cases: comorbid depression (BÄK et al., 2017, high quality) or chronic pain [tricyclics only] (Glocker et al., 2018, low quality)
Anticonvulsants/Antiepileptics including gabapentin, pregablin, carbamazepine, topiramat	5(5)	**Against:** 1x **XX**; 1x **X**; 1x X*	**Against:** 1x X* **Mixed:** 1x **XX** & **O‐** & **O**	Strong AGAINST	
Muscle relaxants including diazepines/benzodiazepines	5(4)	**Against**: 1x **XX** **Mixed:** 1x **XX** & **X** & **O+**	**Against**: 2x X* **Mixed:** 1x **//** & **O**	Strong AGAINST WITH EXCEPTIONS	For specific cases: non‐specific LBP where non‐drug and non‐opioid treatments ineffective (BÄK et al., 2017, high quality); 2^nd^ line medication for acute non‐specific LBP (Regione Toscana, 2015, Low quality)
Spinal injections [for non‐specific LBP]	6(5)	**Against:** 1x **XX;** 1x X*	2x X*, 2x **O**	Strong AGAINST	
Traction	6(6)	**Against:** 2x **XX**; 1x X*	**For:** 1x /* **Against:** 1x **O‐** **Open**: 1x **O**	Strong AGAINST	
Electrotherapy including laser therapies, TENS, PENS, shortwave diathermy, US, ultra‐shortwave, inferential, magnetic field, electromagnetic, light therapy, shockwave, electrostimulation	6(6)	**Against:** 2x **XX**; 1x X*	**Against:** 1x **O‐** **Mixed:** 1x /* & X*; 1x **XX** & **O‐** & **O**	Strong AGAINST	
Orthoses including belts, corsets, foot orthotics, insoles, rocker shoes, pull‐ups, walking stick, elbow crutches and bands	6(6)	**Against:** 2x **XX**; 1x X*	**For:** 1x /* **Against:** 1x **X** **Mixed:** 1x **XX** & **O‐** & **O**	Strong AGAINST	
Imaging	9(6)	**Against**: 3x **X** **Mixed**: 1x **XX** & **//**	**Against**: 1x **XX**; 4x X*	Strong AGAINST WITH EXCEPTIONS	Except: in cases of red flags
**MIXED**					
NSAIDs	9(7)	**For:** 2x **/**; 1x **/** & **O+** **Against:** 1x **X**	**For:** 4x /*; 1x **//** & **O**	Inconsistent	
Opioids (including tramadol) +/‐ paracetamol (or NSAIDs)	8(6)	**Mixed:** 1x **//** & **X**; 1x **O+** & **X**; 1x **/** & X* **Against:** 1x **X**	**For:** 3x /* **Mixed:** 1x **//** & **O+** & **O**	Inconsistent	Generally, ‘Against’ for chronic LBP (unless severe limitations) and ‘For’ where other analgesics ineffective, contraindicated, or not tolerated.
Topical medications/NSAIDS	3(3)	**Against**: 1 x **XX**	**For:** 2x /*	Inconclusive	
Spinal epidural steroid injection	5(5)	**For:** 1x **/** **Mixed**: 1x **/** & X* **Against**: 1x **X**	**For:** 1x /* **Mixed**: 1x **O+** & **O**	Inconsistent	
Other injections including intravenous, intramuscular, infiltration of trigger points and ligaments, intradiscal infiltration, prolotherapy, Botulium toxin	2(2)	1x **XX**	1x **O**	Inconclusive	
Thermotherapy including local heat, hot/cold compresses, baths, sauna	5(4)	**Mixed:** 1x **O+** & **X**	**For:** 2x /* **Open:** 1x **O** **Mixed**: 1x **//** & **O**	Inconsistent	
Manual therapy including mobilisation, manipulation and soft‐tissue techniques	8(6)	**For:** 2x **/** **Mixed:** 1x **XX & O**	**For:** 1x /* **Against:** 2x X* **Mixed:** 1x **XX** & **O**; 1x (**XX** & **O‐** & **O**) & (**//** & **O**)	Inconsistent	
Postural therapies e.g. Alexander therapy, postural re‐education	3(3)	**Open:** 2x **O**	**For:** 1x /*	Inconclusive	
Acupuncture	5(4)	**For**: 1x **O+** **Against**: 1x **X**; 1x X*; 1x **O‐** **Open:** 1x **O**		Inconsistent	
**Single guideline recommendation**					
**FOR**			**AGAINST**		
Analgesics (general)			Antibiotics		
Metamizol			Flurpirtin		
Collaborate with company doctor, company physical therapist or occupation health and safety service			Uridine monophosphate (UMP)		
CAM (general *ie* acupuncture and TCM, phytotherapy, homeopathy, manual therapies)			Kinesiotaping		
Referral to family doctor			Shock‐absorbing or anti‐fatigue flooring		
Referral to manual therapist			**MIXED**		
Referral to family doctor, company doctor and/or psychologist			Steroids		
Referral for specialist assessment			Progressive muscle relaxation		
Bioptron lamps (SC)			Phytotherapeutics		
Ledotherapy lamps (SC)			Topical phytotherapeutics		
Infra‐red raditation (SC)			**OPEN**		
Bath salts with mud extracts or special water‐pearling inserts or even ozone (SC)			Spa treatments		
Magnetic mattress (SC)			Ozone therapy		
			Medullary stimulations		
			'Taking it slowly/easy'		

Psychological therapies are mainly recommended for subgroups of patients with increased psychosocial risks, mood problems, or more complex, persistent back pain; whereas referral for surgery is only supported for cases with signs of specific pathology.

Overall, guidelines recommended strongly against the use of more than a couple of days bedrest for patients with low back pain. Referral for imaging is only supported for those with red flags, such as increased risk of fracture, infection, (metastatic) cancer, neurological emergencies including cauda equina syndrome, aortic aneurysm or systemic inflammatory arthritis (detailed in Appendix S7), or deterioration of symptoms. And although mixed, moderate to strong recommendations were also given against the use of paracetamol, anti‐depressants, anticonvulsants and muscle relaxants; spinal injections for non‐specific LBP; traction; orthoses and a range of applications (e.g. electrotherapies, shortwave, laser).

Recommendations were inconsistent or inconclusive with respect to medication (NSAIDs, opioids; topical); epidural steroid and other injections; acupuncture and manual, postural and thermotherapies.

### 3.6 Comparison of CPG recommendations for neck and low back pain

In order to examine the consistency of CPG recommendations across neck and low back pain, overall strengths of recommendation for each identified intervention (see Tables 3&4), were assessed (Table 5). Despite a larger body of evidence for the effectiveness of treatment for back pain and a larger number of back pain guidelines, recommendations were generally consistent for neck and back pain (Table 5), in particular regarding support for the use of advice and education, reassurance, certain oral and topical pharmacologic treatments (with the exception of paracetamol), exercise interventions, manual therapy when combined with active treatment and psychological interventions. Guidance was also consistent in terms of the limited use of imaging (only for patients with red flags or where imaging is likely to change management), and recommendations against the use of bed rest, orthoses, traction and a range of modalities (laser therapy, electrotherapy, shortwave).

**Table 5:** Consistency of recommendations across low back pain vs neck pain guidelines

Intervention	Low Back Pain		Neck Pain	
	No. guidelines (countries)	Overall strength of recommendation	No. guidelines (countries)	Overall strength of recommendation
Reassurance (advice)	4(4)	Weak FOR	3(3)	Weak FOR
Advice and Education (advice)	10(8)	Strong FOR	5(5)	Weak FOR
Remain active (advice)	9(6)	Strong FOR	2(2)	Weak FOR
Encourage physical exercise (advice)	7(6)	Weak FOR	3(3)	Weak FOR
Continue/return to work (advice)	2(2)	Weak FOR	1(1)	(For)
Bed rest (advice)	6(4)	Strong AGAINST WITH EXCEPTIONS	1(1)	(Against)
				
Analgesics incl. for neuropathic pain	1(1)	(For)	2(2)	Weak FOR
Paracetamol	8(6)	Moderate AGAINST	2(2)	Weak FOR
NSAIDs	9(7)	Inconsistent	4(3)	Weak FOR
Opioids (including tramadol) +/‐ paracetamol (or NSAIDs)	8(6)	Inconsistent	2(1)	Weak FOR
Antidepressants	6(5)	Strong AGAINST WITH EXCEPTIONS		
Anticonvulsants/Antiepileptics	5(5)	Strong AGAINST		
Muscle relaxants	5(4)	Strong AGAINST WITH EXCEPTIONS		
Topical medications incl. NSAIDS	3(3)	Inconclusive	2(2)	Moderate FOR
Spinal injections [for non‐specific LBP]	6(5)	Strong AGAINST		
Spinal epidural steroid injection	5(5)	Inconsistent	1(1)	(For)
Other injections	2(2)	Inconclusive		
				
Thermotherapy	5(4)	Inconsistent	2(2)	Inconclusive
Manual therapy	8(6)	Inconsistent	5(4)	Inconsistent
Manual therapy combined with other treatment	4(3)	Moderate FOR	3(3)	Moderate FOR
Exercise programs/therapy	9(6)	Strong FOR	5(5)	Moderate FOR
Exercise therapy combined with other treatment			2(2)	Moderate FOR
Group exercise programmes/back schools	3(3)	Moderate FOR		
Postural therapies	3(3)	Inconclusive		
Traction	6(6)	Strong AGAINST	3(3)	Inconclusive
Electrotherapy	6(6)	Strong AGAINST	4(4)	Inconclusive
Orthoses	6(6)	Strong AGAINST	4(4)	Inconclusive
Acupuncture	5(4)	Inconsistent	4(3)	Inconsistent
Psychological therapies	4(3)	Strong FOR SPECIFIC SUBGROUPS	3(3)	Weak FOR SPECIFIC SUBGROUPS
Psychological therapies combined with other treatment	2(2)	Moderate FOR		
Multidisciplinary treatment	7(5)	Strong FOR SPECIFIC SUBGROUPS	2(2)	Weak FOR SPECIFIC SUBGROUPS
				
Work‐based interventions	3(3)	Moderate FOR		
Return to work programmes	3(3)	Strong FOR		
				
Imaging	9(6)	Strong AGAINST WITH EXCEPTIONS	2(2)	Inconclusive
To surgeon/surgery	8(6)	Strong FOR SPECIFIC SUBGROUPS		Appendix S1 Appendix S2 Appendix S3 Appendix S4 Appendix S5 Appendix S6 Appendix S7

Referral for imaging or surgical intervention, bed rest, antidepressant and muscle relaxant medications, psychological or multidisciplinary interventions are recommended for specific subgroups of patients (FOR ‘SPECIFIC SUBGROUPS’ or AGAINST ‘WITH EXCEPTIONS’ in Table 5).

## 4 DISCUSSION

In this review, we have systematically identified, synthesized and graded 17 European clinical guidelines relating to the management of NLBP. On the basis of the quality of the evidence we have identified a short list of treatment options recommended for the management of NLBP (see Table 5). This information is aimed to provide clinicians, healthcare managers, funders, policymakers and researchers with a comprehensive summary of the current consensus from clinical guidelines across Europe on the management of NLBP.

The guidelines included in our review came from eight European countries (UK, Germany, France, Italy, Denmark, Poland, Belgium and the Netherlands). Eleven of them addressed low back pain, five neck pain and one both LBP and neck pain. Data extraction showed considerable variation in guideline development processes with seven guidelines (5 back, 2 neck) considered as high quality, based on their development rigour, strong stakeholder involvement and the applicability of their recommendations.

For ***neck pain***, high‐quality guidelines consistently recommended the following evidence‐based treatment options: reassurance, advice and education (including to remain active and exercise), manual therapy in combination with other treatment, referral for exercise therapy/programme and a range of oral analgesics and topical medications, plus psychological therapies or multidisciplinary treatment for specific subgroups of patients. There was no strong evidence for use across Europe (as shown in Table 3). In contrast to the recommendations for low back pain, the neck pain guidelines included the use of painkillers such as paracetamol, NSAIDs (for acute pain only), opioids (for acute pain only) and neuropathic pain medication. However, these were only based on ***weak evidence*** (meaning the recommendations were based on expert opinion only from high‐quality guidelines, and/or multiple low‐quality guidelines) and it should be noted that these medications are no longer consistently recommended for low back pain within the recent European guidelines. In fact, for ***low back pain*** the guidelines recommended entirely non‐pharmacological treatments, additionally including work‐based interventions, advice/programmes to return to work and surgical intervention for specific subgroups. These recommendations were based on stronger evidence than those for neck pain.

In relation to previous literature, the findings of this review summarising the consensus from European guidelines, are consistent with recommendations in The Lancet back pain series (Foster et al., 2018) which advocated for greater use of non‐pharmacological options for patients with back pain. The treatment options identified in this study are also broadly similar and consistent with two recent systematic reviews of clinical practice guidelines for musculoskeletal pain (Lin et al., 2020) and back pain (Oliveira et al., 2018) which identified similar key management recommendations (patient information, physical activity advice, return to work interventions, exercise interventions), although Oliveira et al., additionally identified antidepressants (for chronic LBP), NSAIDs and weak opioids for short periods of time (for acute LBP) to be frequently recommended across guidelines.

Recommendations from the European guidelines included in our review contrast notably with a systematic review of non‐invasive treatments for low back pain conducted to inform the American College of Physicians Clinical Practice Guideline (Chou et al., 2016) which not only recommended three medication options (NSAIDs, opioids, duloxetine) with moderate to strong evidence (Chou, et al., 2017b), but also included acupuncture within a group of 5 recommended non‐pharmacological options (superficial heat, multi‐disciplinary rehabilitation, acupuncture, exercise and manual therapy) (Chou, et al., 2017a).

Many of the European guidelines included treatment recommendations related to patient subgroups: psychological therapies, multi‐disciplinary treatment and referral for surgery were recommended for specific subgroups only; and very strict indications (strong recommendation against with exception given for bed rest, anti‐depressants and muscle relaxants). However, it was notable that clear assessment criteria to facilitate clinician decision making about when to use these treatment options for specific patient subgroups were largely lacking. Similar to Lin et al. who highlighted that guidelines for patients with thoracic pain are lacking (Lin et al., 2020), we only identified one (low quality) guideline (Kassolik et al., 2017) that specifically addressed thoracic pain. We would also highlight that most guidelines lacked detail about the specific dose, duration and other detail around the delivery of the recommended treatments. For example, there was little clarity on the delivery of physical exercise or the recommended components of patient education or reassurance.

### Strength and limitations

The strength of this review is that it provides a helpful overall summary of the treatment and referral recommendations from recent European guidelines for NLBP. This overview enabled us to identify treatment options that have been consistently recommended across eight different countries and can therefore be considered to have broad European consensus. To facilitate the rigour of this evidence summary, we pre‐specified inclusion and exclusion criteria for screening, quality‐appraised guidelines using the AGREE II checklist, and devised a set of clear criteria to summarize and synthesize the direction and strength of recommendations across guidelines. Further strengths included independent assessment of eligibility for inclusion, data extraction and appraisal of the quality of guidelines, and a standardized approach to synthesizing evidence.

The guidelines included in our systematic review predominantly originate from northern and western European countries (except for the Italian guidelines), which can be considered a limitation. This may be partly explained by fewer guidelines being produced in southern or eastern Europe, but also by the fact that we only included guidelines published in the past 7 years. While focusing on contemporary guidelines (2013 onwards) ensured that we identified the most relevant treatment options for current practice, we acknowledge that this meant that some earlier European guidelines, were not included. However, for the purposes of this review, we felt it was important to exclude guidelines that may not be based on up‐to‐date evidence of effectiveness. Although we included guidelines written in any European language, one limitation was that we were not able to carry out independent data extraction and quality appraisal by a second reviewer for guidelines not available in English. However, for most of these guidelines, the reviewer had the advantage of being involved in data extraction for English language guidelines, which helped to ensure consistency of data extraction and interpretation.

Only seven CPGs (41%) were considered to be of overall high quality, with limitations mainly related to rigour of development (e.g. use of transparent methods to link evidence to recommendations, or processes to gain consensus regarding the strength of recommendations); and to applicability with few guidelines providing guidance on how to apply recommendations or taking into account practical and financial implications of their recommendations. Variation in the methods used to grade evidence and agree the strength of recommendations may potentially explain some of the variability in treatment recommendations across guidelines. We tried to incorporate quality as well as consistency in our synthesis of CPGs, aiming to arrive at a transparent and systematic approach for summarizing and grading recommendations across guidelines.

Future work within the Back‐UP research project will embed these evidence‐based treatment options in an accessible clinician decision‐support tool for first contact clinicians, aiming to offer patients with NLBP treatment options better matched to the risk of persistent pain and disability.

## 5 CONCLUSION

In conclusion, this systematic review identified seventeen contemporary clinical guidelines regarding NLBP (5 neck; 11 low back; 1 both) from 8 European countries, of which seven were considered high quality. Recommendations were notably consistent for neck and low back pain, despite the larger evidence base and more guidelines for the latter. The implications of this review are that clinicians have a broad range of mostly non‐pharmacological evidence‐based treatment options to consider for their patients with NLBP. These include some treatments which are a) potentially applicable to all patients such as advice and education and b) those applicable only to certain patient subgroups (e.g. referral to surgery).

## CONFLICTS OF INTEREST

The authors have no conflicts of interest to declare.

## AUTHORS’ CONTRIBUTION

GM, DvdW and JH designed the study protocol with input from NC. NC designed the literature search with input from DvdW. NC, GM and DvdW performed the study selection with input from LM. NC, GM, LM, SS and GW‐J carried out data extraction and quality assessment. NC, JH, SS and DvdW carried out the analysis and interpretation of the data. NC and JH drafted the manuscript. All authors critically revised the manuscript for intellectual content and read and approved the final manuscript.

## Supporting information

Appendix S1

Appendix S2

Appendix S3

Appendix S4

Appendix S5

Appendix S6

Appendix S7
