# Supplementary material for: Evidence‐based treatment recommendations for neck and low back pain across Europe: A systematic review of guidelines
Source: Eur J Pain. 2020 Nov 12;25(2):275–95. doi: 10.1002/ejp.1679 (PMC7839780; doi:10.1002/ejp.1679)
Supplement: Supplementary file 1 — Appendix S1 [file EJP-25-275-s001.docx]

Supporting Information Appendix S1: MEDLINE (Ovid) Search strategy

The following table is an explanation of the symbols used in the search strategy below.

/ indicates an index term (MeSH heading)

exp before an index term indicates that all subheadings were selected

.ab. indicates a search for a term in the abstract only

.ti,ab,kw. indicates a search for a term in the title, abstract and keyword

.ti,ab,kw,kf. indicates a search for a term in the title, abstract, keyword and word in keyword

$ at the end of a term indicates that this term has been truncated

$*n* at the end of a term indicates that this term has been truncated

adj indicates a search for two terms where they appear adjacent to each another

adj*n* indicates a search for two terms where they appear within *n* words of each another

| 1 | exp Back Pain/ |
| --- | --- |
| 2 | dorsalgia.ti,ab,kw. |
| 3 | (backache or back ache).ti,ab,kw. |
| 4 | ((back or lumb$) adj3 pain).ti,ab,kw. |
| 5 | ((spine or spinal) adj3 pain).ti,ab,kw. |
| 6 | coccyx.ti,ab,kw. |
| 7 | coccydynia.ti,ab,kw. |
| 8 | exp Sciatic Neuropathy/ |
| 9 | sciatica.ti,ab,kw. |
| 10 | spondylosis.ti,ab,kw. |
| 11 | lumbago.ti,ab,kw. |
| 12 | back disorder$.ti,ab,kw. |
| 13 | Neck Pain/ |
| 14 | ((neck or cervical) adj3 pain).ti,ab,kw. |
| 15 | (neck adj3 ache).ti,ab,kw. |
| 16 | neckache.ti,ab,kw. |
| 17 | cervicalgia.ti,ab,kw. |
| 18 | cervicodynia.ti,ab,kw. |
| 19 | Whiplash Injuries/ |
| 20 | whiplash.ti,ab,kw. |
| 21 | neck disorder$.ti,ab,kw. |
| 22 | intervertebral disc displacement/ |
| 23 | ((disk$ or disc$) adj3 (herniat$ or prolapse$ or slipped)).ti,ab,kw. |
| 24 | radiculopathy/ |
| 25 | radicul$.ti,ab,kw. |
| 26 | or/1-25 |
| 27 | practice guideline/ |
| 28 | Health Planning Guidelines/ |
| 29 | guideline$1.ti,kw,kf. |
| 30 | guidance.ti,kw,kf. |
| 31 | standards.ti,kw,kf. |
| 32 | ((practice or treatment$ or clinical) adj standard).ti,kw,kf. |
| 33 | recommendation$1.ti,kw,kf. |
| 34 | ((practice or treatment$ or clinical) adj3 consensus).ti,kw,kf. |
| 35 | Consensus Development Conference.pt. |
| 36 | (practice adj (guideline$1 or guidance or standard$1 or recommendation$1)).ab. |
| 37 | (clinical adj (guideline$1 or guidance or standard$1 or recommendation$1)).ab. |
| 38 | (treatment$ adj3 (guideline$1 or guidance or standard$1 or recommendation$1)).ab. |
| 39 | (CPG or CPGs).ti,kw,kf. |
| 40 | Critical Pathways/ |
| 41 | position statement$1.ti,ab,kw,kf. |
| 42 | policy statement$1.ti,ab,kw,kf. |
| 43 | (practice adj3 parameter$1).ti,ab,kw,kf. |
| 44 | (((critical or clinical or practice) adj3 (path$1 or pathway$1 or protocol$1)) and (guideline$1 or guidance or standard$1 or recommendation$1)).ab. |
| 45 | ((care adj3 (path$1 or pathway$1 or map$1 or plan or plans)) and (guideline$1 or guidance or standard$1 or recommendation$1)).ab. |
| 46 | ((care adj3 standard$1) and (guideline$1 or guidance or recommendation$1)).ab. |
| 47 | or/27-46 |
| 48 | 26 and 47 |
| 49 | limit 48 to yr="2013 -Current" |
