# Supplementary material for: Evidence‐based treatment recommendations for neck and low back pain across Europe: A systematic review of guidelines
Source: Eur J Pain. 2020 Nov 12;25(2):275–95. doi: 10.1002/ejp.1679 (PMC7839780; doi:10.1002/ejp.1679)
Supplement: Supplementary file 2 — Appendix S2 [file EJP-25-275-s002.docx]

Supporting Information Appendix S2 – Classification of recommendations

|  | Symbol - Classification | Definition | Possible terminology and associated guidelines |
| --- | --- | --- | --- |
| FOR | // - Should do | Recommendations that are strongly endorsed and should be applied in all circumstances unless there is reason not to. Based on strong evidence reporting clinically relevant benefit that outweighs risk. | - ‘strong’ (Sundhedsstyrelsen, 2015, 2016a-c), with wording ’should’ (Pohl et al., 2018; Bier et al., 2016), or ’offer’ (NICE, 2016; van Wambeke et al., 2017). - Recommendation strength A (Monticone et al., 2013; Regione Toscana, 2015; SFMT, 2013), with wording ‘do’ (BÄK et al., 2017) - Level 1 (Staal et al., 2017) |
|  | / - Could do | Recommendations where there are doubts as to whether the intervention should always be applied and therefore implementation should be carefully considered. Based on consistent but weak evidence reporting clinically relevant benefit that outweighs risk. | - ‘weak’ (NICE, 2016; Pohl et al., 2018; Sundhedsstyrelsen, 2015, 2016a-c; van Wambeke et al., 2017), with wording ‘can be used’ (Bier et al., 2016) - Recommendation strength B (Regione Toscana, 2015; Monticone et al., 2013) - Recommendation B/’weak’ (BÄK et al., 2017) - Recommendation strength B or C (SFMT, 2013) - Level 2 (Staal et al., 2017) |
|  | /* - For (generic) | Recommendation in favour of intervention, where no formal grading system has been used. | - For (Bons et al., 2017; Glocker et al., 2018; Kassolik et al., 2017; Schaafstra et al., 2015) |
| OPEN | O [O+/O-] – Open | Where no recommendation for or against an intervention can be made, because of an insufficient or inconsistent/conflicting evidence-base.  Where expert opinion was then employed to indicate in favour of or against an intervention, this is indicated with a plus (O+) or minus (O-), respectively. | - Open (Pohl et al., 2018; BÄK et al., 2017) - ‘no recommendation’ (NICE, 2016; van Wambeke et al., 2017) - ‘no advice either way’ (Glocker et al., 2018) - Recommendation strength C (Monticone et al., 2013; Regione Toscana, 2015) - ‘unclear evidence’ (SFMT, 2013) - Level 3 or 4 (Staal et al., 2017) - For: expert opinion (van Wambeke et al., 2017); Good practice (Sundhedsstyrelsen, 2015, 2016a-c ); Open: may be used (BÄK et al., 2017); ‘may be used’ (Bier et al., 2016); GDG agreed (NICE, 2016); ‘Seems to be effective’ (SFMT, 2013); Recommendation strength A* (Regione Toscana, 2015) - Not good practice (Sundhedsstyrelsen, 2015, 2016c); ‘Evidence of ineffectiveness’ (Regione Toscana, 2015) |
| AGAINST | X* - Against (generic) | Recommendation against an intervention, where no formal grading system used, or applied to recommendations against. | - Against (Bier et al., 2016; Bons et al., 2017; Glocker et al., 2018; Kassolik et al., 2017; Schaafstra et al., 2015) - ‘Do not offer’ (NICE, 2016) |
|  | X – Should not do | Recommendations where there are doubts as to whether the intervention should not always be applied and should be carefully consideration. Based on consistent but weak evidence reporting no clinically relevant benefit, and/or harms that outweigh benefit. | - ‘weak’ (Pohl et al., 2018; Sundhedsstyrelsen 2015, 2016a-c) with wording ‘do not routinely offer’ (van Wambeke et al., 2017); - Recommendation strength B (BÄK et al., 2017); Pohl), - Recommendation strength B or C (SFMT, 2013) - Recommendation D (Monticone et al., 2013) - Level 2 (Staal et al., 2017) |
|  | XX - Definitely do not do | Recommendations where there is strong evidence of no clinically relevant benefit and/or harms outweighing benefits. | - ‘strong’ with wording ’do not offer’ (van Wambeke et al., 2017), or ‘should not’ (Pohl et al., 2018) - Recommendation strength A (SFMT, 2013; Pohl et al., 2018) with wording ‘do not’ (BÄK et al., 2017) - Recommendation E/’strongly discouraged’ (Monticone et al., 2013) - Level 1 (Staal et al., 2017) |
