# Supplementary material for: Evidence‐based treatment recommendations for neck and low back pain across Europe: A systematic review of guidelines
Source: Eur J Pain. 2020 Nov 12;25(2):275–95. doi: 10.1002/ejp.1679 (PMC7839780; doi:10.1002/ejp.1679)
Supplement: Supplementary file 3 — Appendix S3 [file EJP-25-275-s003.docx]

Supporting Information Appendix S3: Basic characteristics of included clinical practice guidelines

| Guideline ID, publication date | Country | Organisation(s) | Target Population | Diagnostic classification (incl./excl.) |
| --- | --- | --- | --- | --- |
| Neck pain | | | | |
| Bier *et al.,* 2016  [Nov-16] | Netherlands | Royal Dutch Society for Physical Therapy | Neck pain (Grades I-IV); Physical and manual therapists. | Neck pain defined as "an unpleasant sensory and emotional experience that is associated with actual or potential tissue damage" in the neck region (from superior nuchal line to scapular spine), potentially accompanied by pain in the head, shoulder, and/or arm. Severity is classified into Grades I-IV. |
| Monticone *et al.,* 2013 | Italy | The Italian Society of Physical and Rehabilitation Medicine (SIMFER) | Neck pain; Specialists in physical and rehabilitation medicine, or involved in treating spinal disorders (orthopaedists, neurologists, rheumatologists), rehabilitation team members (occupational therapists, physical therapists, psychologists, general practitioners), and patients and their families. | Neck pain |
| Pohl *et al.,* 2018 | Germany | German Association of Scientific Medical Societies | Degenerative cervical radiculopathy (CR); All health professionals involved in the management of CR. | Degenerative cervical radiculopathy, confirmed by imaging (MRI) |
| Sundhedsstryrelsen 2015  [20-May-15, ver 1.0] | Denmark | Danish Health Authority | Patients >18 years; Pain <12 weeks; Clinical signs of cervical radiculopathy caused by disk herniation or degenerative changes; Primary care | Recent onset cervical radiculopathy, excluding radiculopathy caused by cancer, infections, traumas, circulatory insufficiency, osteoporosis or arm pain that does not stem from changes in the cervical spine.  *nb*: no distinction is made between cervical radiculopathy caused by disc herniation and other degenerative conditions, as treatment is usually undertaken without existing imaging. |
| Sundhedsstryrelsen 2016c  [29-Nov-16, ver 1.0] | Denmark | Danish Health Authority | Adult patients; Pain <12 weeks located in the neck region; Primary care | Recent onset neck pain, excluding radiculopathy, traumatic neck pain, and specific causes of neck pain including cancer, inflammatory muscle and joint disease, infections, circulatory insufficiency, and osteoporosis. |
| Low back pain | | | | |
| BÄK, *et al.,* 2017  (2^nd^ edition, ver. 1) [update from Oct-15] | Germany | 30 health organisations, including German Medical Association, National Association of Statutory Health Insurance Physicians, and Scientific Medical Societies | Low back pain in all settings; All medical and allied health professionals, adults with LBP and their relatives, healthcare decision-makers, and members of the general public. | Nonspecific low back pain: pain in the back between costal arch and gluteal fold, with or without radiating pain, but excluding radiculopathy, tumours, infection, and fractures. |
| Bons *et al.,* 2017  (2^nd^ revision)  [Feb-17; update from 2005] | Netherlands | Dutch College of General Practitioners | Low back pain; General Practitioners, primary care. | Non-specific low back pain: back pain between the lower ribs and buttocks, where no specific physical cause can be identified, thus excluding malignancy, vertebral fracture, axial spondyloarthritis, and lumbosacral radicular syndrome. |
| Glocker *et al.*, 2018  [11-Jan-18] | Germany | German Society for Neurology in collaboration with associations for neurosurgery, orthopaedics and orthopaedic surgery | Adults with acute back/leg pain due to lumbar radiculopathy (lumbar nerve compression or damaged), presenting in 1° or 2° care, or rehabilitation | Lumbar radiculopathy (established pathology, not radicular or referred pain only). Imaging required in patients with symptoms and neurological signs indicating radiculopathy. |
| National Institute for Health Care Excellence (NICE), 2016  [30-Nov-16] | UK | National Institute for Health and Care Excellence (NICE) | Low back pain and sciatica; Individuals ≥ 16 years old; Primary and secondary care settings (invasive and non-invasive treatment options. | Low back pain including discogenic pain, degenerative disc disease, lumbar disc herniation, secondary to lumbar degenerative disease, and facet joint pain.  Sciatica including sciatica/lumbago, radicular pain/radiculopathy, pain radiating to the leg, neurogenic claudication, and nerve root compression/irritation.  Exclusion: pregnancy-related back pain, spondylolisthesis, osteoarthritis, sacroiliac joint dysfunction, adjacent-segment disease, failed back surgery syndrome, and serious underlying pathologies. Mixed populations, unless data for people with low back pain only can be extractable. |
| Regione Toscana, 2015  [update from 2005] | Italy | Consiglio Sanitario Regionale, Regione Toscana | Back pain | Back pain including acute non-specific low back pain; acute radiculopathy (sciatica or cruralgia); persistent acute back pain, subacute and chronic non-specific back pain, and specific chronic low back pain. |
| Schaafstra *et al.* 2015 (2^nd^ revision)  [update from 2005] | Netherlands | Dutch College of General Practitioners | Lumbosacral radicular syndrome; General practitioners, primary care | Lumbosacral radicular syndrome, defined as radiating pain in one buttock or leg, with or without other stimulation phenomena (paraesthesia) and neurological deficits (hypaesthesia/hypalgesia, paresis, reduced reflexes), which is suggestive of stimulation of one (sometimes two) specific lumbosacral nerve root(s). |
| Société Française de Médecine du Travail (SFMT) (2013)  [21-Oct-13] | France | French Society of Occupational Medicine. Partners: National Institute of Research and Security, French Society of Rheumatology (Spine section), National Institute of Health and Medical Research, French Society of Physical Medicine and Rehabilitation, Department of Occupational Health and Health Education, University of Liège. | Adult workers exposed to manual handling and lifting; Lumbar spine | Recommendations relate to the lumbar spine (though some of the studies reviewed relate to other areas) |
| Staal B. *et al*. 2017  [update from 2013] | Netherlands | Royal College of Physiotherapists together with professional organisation for manual therapists | Low back pain; Physiotherapists and manual therapists | Low back pain, including non-specific LBP and pain due to specific spinal pathology |
| Sundhedsstryrelsen 2016a  [20-Jun-16, ver 1.0] | Denmark | Danish Health Authority | Patients>16 years; Pain <12 weeks regardless of prior episodes; Localised from Th12 to inferior glut fold with or without leg pain; Primary Care | Non-specific low back pain with or without leg pain, excluding lumbar radiculopathy. |
| Sundhedsstryrelsen 2016b  [19-Jan-16, ver 1.0] | Denmark | Danish Health Authority | Patients >18 years; Pain <12 weeks; Clinical signs of lumbar radiculopathy with or without MRI verification; Primary care | Recent onset lumbar radiculopathy whether due to disc herniation or facet joint (or unclear from MRI), but excluding radiculopathy due to other causes e.g. tumours, spinal stenosis, spondylolisthesis and diabetes mellitus. |
| van Wambeke *et al.* 2017  [27-Nov-17, 2nd ed] | Belgium | Belgian Health Care Knowledge Centre | Low back pain and sciatica; Individuals ≥ 16 years old; Primary and secondary care settings (invasive and non-invasive treatment options. | Low back pain defined as pain between the bottom of the rib cage and the buttock creases, without serious underlying cause (red flags), and radicular pain (incl. neurogenic claudication). Covers acute (0-6 weeks), sub-acute (6-12 weeks) and chronic (from 12 weeks) phases.  Exclusion of serious spinal pathology (infection, malignancy and fractures), inflammatory conditions (ankylosing spondylarthritis), potentially serious neurological sequelae of sciatica (progressive neurological deficit and cauda equina syndrome), pregnancy-related back pain, sacroiliac joint dysfunction, adjacent-segment disease, failed back surgery syndrome, and spondylolisthesis. |
| Neck and low back pain | | | | |
| Kasssolik *et al.,* 2017 | Poland | Polish Society of Physiotherapy, Polish Society of Family Medicine and College of Family Physicians in Poland | Back pain syndromes; Primary care. | Cervical back pain syndrome: as defined by the International Association for the Study of Pain *i.e.* neck pain below the nuchal line and above the line marked by the transverse line running through the 1^st^ spinous process of the thoracic spine and laterally through the sagittal plane adjacent to lateral surface of the neck.  Thoracic back pain syndrome: Pain experienced in the upper and middle back of the torso between the Th1–Th12 vertebrae.  Lumbar-sacral back pain syndrome *i.e.* low back pain (LBP): below 12th rib and above lower gluteal folds, associated with possible radiation to the lower limbs. |
