# Supplementary material for: Evidence‐based treatment recommendations for neck and low back pain across Europe: A systematic review of guidelines
Source: Eur J Pain. 2020 Nov 12;25(2):275–95. doi: 10.1002/ejp.1679 (PMC7839780; doi:10.1002/ejp.1679)
Supplement: Supplementary file 4 — Appendix S4 [file EJP-25-275-s004.docx]

Supporting Information Appendix S4 - Development and implementation methods used in clinical practice guidelines

| Guideline ID | Multi-disciplinary group committee or single profession? | Date or timeframe for future revision | How was level of evidence determined and/or strength of recommendation determined | Consensus (details) | includes recommendations regarding (Y/N): | | | | | | |
| --- | --- | --- | --- | --- | --- | --- | --- | --- | --- | --- | --- |
|  |  |  |  |  | Evaluation of flags | | | Diagnosis | Planning of care | Practitioner education | Organisation and policy |
|  |  |  |  |  | red | yellow | blue/ black |  |  |  |  |
| Neck pain | | | | | | | | | | | |
| Bier et al., 2016 | 9 authors in total; 8 describe themselves as physiotherapists and/or manual therapists, 1 describes themselves as an epidemiologist. 4 of the physiotherapists/manual therapists additionally describe themselves as epidemiologists; 2 physiotherapists/manual therapists additionally describe themselves as policy advisors. | Not reported | GRADE (High, Moderate, Low, Very low)  Based on: High quality evidence; Low quality evidence; and expert opinion. Recommendation wording ranging from: recommended to use (should be used - A); can be used - B; may be used - 0; recommended against | Specific interventions searched for; best evidence sought through specific guidelines and Cochrane databases; additional electronic searches also conducted; quality appraisal conducted | Y | Y | Y | Y | Y | N | N |
| Monticone et al., 2013 | Not reported | Not reported | Evidence graded: I (highest) – VI (lowest)  Strength of recommendation: A (strongly recommend) – E (strongly discourage) | Not reported, it is unclear whether this was undertaken | Y | N | N | Y | Y | N | N |
| Pohl et al., 2018 | Multidisciplinary: experts from neurology, orthopaedics, physiotherapy, emergency medicine, trauma surgery, neurosurgery, pain medicine, occupational therapy, and patient organisation. | Valid for 3 years (until 2021) | Recommendations graded: "soll" (should = strong); "sollte” (may = weak); or "kann" (open) | Consensus achieved through Nominal Group Technique (2 meetings and Delphi survey). Recommendations adopted with at least 88% agreement | Y | Y | N | Y | Y | N | N |
| Sundhedsstryrelsen, 2015 | Development/Working group consisting of 10 interdisciplinary clinicians. Reference group consisting of 11 interdisciplinary clinicians. Secretariat consisting of 5 persons from Danish Health Authority | Every 3 years, or if new evidence emerges | Overall level of evidence (high, moderate, low, very low) and strength of recommendations (Strong, weak, good practice) determined using GRADE | The guideline used the Danish Health Authorities recommendations that are based on the GRADE system | N | N | N | N | Y | Y | N |
| Sundhedsstryrelsen, 2016c | Development/Working group consisting of 12 interdisciplinary clinicians. Reference group consisting of 11 interdisciplinary clinicians. Secretariat consisting of 3 persons from Danish Health Authority | Every 3 years, or if new evidence emerges | Overall level of evidence (high, moderate, low, very low) and strength of recommendations (Strong, weak, good practice) determined using GRADE | The guideline used the Danish Health Authorities recommendations that are based on the GRADE system | N | N | N | N | Y | Y | N |
| Low back pain | | | | | | | | | | | |
| BÄK et al., 2017 | Multidisciplinary: Aimed at all medical professions involved in the detection, diagnosis and treatment of patients with low back pain, treatment-support specialists (e.g. occupational therapy, physiotherapy, psychotherapy, social work); specialty hospitals and departments, acute and rehabilitative hospitals | update planned 5 years after publication | Oxford Centre of Evidence 2011 levels of evidence: level 1 to 5, with type of study design depending on type of research question + AMSTAR score for each review  Recommendations graded using GRADE approach: A (strong), either 'do' or 'do not'; or B (weak), either 'should do' or 'should not do'; or 0 (open): 'may do'. | Multi-part nominal group process with representatives of different healthcare specialties and organisations. Each organisation had one vote. process includes 6 steps: (i) silent review of the guideline manuscript; (ii) opportunity to propose changes to recommendations and grading; (iii) moderator records the judgements and alternative proposals; (iv) preliminary voting on all recommendations; (v) discussion on issues where there is no consensus; (vi) final vote | Y | Y | Y | Y | Y | N | Y |
| Bons et al., 2017 (2^nd^ revision) | Single profession: general practitioners, but aligned with the multidisciplinary (CBO) guidance for non-specific LBP and developed together with national organisations for neurologists, orthopedists, physiotherapist, etc. Patient involvement through meeting with patient organisation. | Not reported | Narrative summaries of the evidence.  Recommendations not graded. | Literature searches conducted by College of GPs. Working group reviews evidence and formulates recommendations. Feedback requested from number of specialists and experts; followed by round of feedback from random sample of (n=50) GPs. | Y | Y | Y | Y | Y | N | Y |
| Glocker et al., 2018 | Multidisciplinary, aimed at neurologists, neursurgeons, orthopedic doctors and surgeons, and for information for rehabilitation physicians and physiotherapists | 2023 | Not used | First round send to panel members for comments; teleconference to discuss disagreements; final draft distributed. Agreement scored on 1-6 scale; but results not reported for each treatment option. | Y | Y | N | Y | N | N | N |
| National Institute for Health Care Excellence (NICE), 2016 | Multidisciplinary group including: neurosurgeon, GP, nurse, psychologist, physiotherapist, osteopath, patients, researchers, orthopaedic surgeon, rheumatologist, Pain medicine consultant, and health economist | Review of evidence base after publication, to establish if it has progressed significantly to alter recommendations and thus warrant update. | Overall level of evidence determined using GRADE (high, moderate, low, very low).  Strength of recommendation based on GRADE. Wording in guideline to reflect strength: 'offer' - strong recommendation (usually where there is clear evidence of benefit); 'consider' - a recommendation for which the evidence of benefit is less certain. | Systematic reviews conducted. Recommendations drafted on the basis of the GDG’s interpretation of the available evidence, or expert opinion (agreed though discussion) where evidence was of poor quality, conflicting or absent.  Recommendation wording was agreed by the GDG taking into consideration the actions health professionals need to take, the information readers need to know, the strength of the recommendation, the involvement of patients (and their carers if needed) in decisions on treatment and care, and consistency with NICE’s standard advice on recommendations about drugs, waiting times and ineffective interventions | Y | Y | N | Y | Y | Y | N |
| Regione Toscana, 2015 | Multi-professional team: doctors (general medicine, orthopaedics, rheumatology), psychologists and psychotherapists | Not reported | Evidence graded: I (highest) – VI (lowest)  Recommendations graded:   - A. Strong; - A*. Strong, where evaluation with RCT not possible, or based on irrefutable clinical experience - B. Doubts as to whether recommendation should always be implemented and should be carefully considered. - C. Substantial uncertainty; where no studies found, or studies report conflicting results. | **Check this is the right information and if so summarise?** | Y | Y | Y | Y | Y | Y | Y |
| Schaafstra et al., 2015 (2^nd^ revision) | Single profession: general practitioners but aligned with the multidisciplinary (CBO) guidance for lumbar radicular syndrome and developed together with national organisations for neurologists, orthopaedists, and physiotherapists. Patient involvement through meeting with patient organisation. | Not stated | Narrative summaries of the evidence.  Recommendations not graded. | Literature searches conducted by College of GPs. Working group reviews evidence and formulates recommendations. Feedback requested from number of specialists and experts; followed by round of feedback from random sample of GPs (*n* = 50). | Y | N | N | Y | Y | N | N |
| Société Française de Médecine du Travail (SFMT), 2013 | Working group consisting of medical practitioners (n=24): specialists in rheumatology, industrial medicine, osteopathy, rehabilitation, physiotherapy, ergonomics, occupational therapy, nursing, physiology, and epidemiology.  Service users also involved.  'Reading group' (n=50) to review the literature: similar composition to working group. | Not stated | Overall level of evidence graded: 1 (highest) to 4 (lowest).  Recommendations graded:  A. level of evidence 1 (highest);  B. level of evidence 2;  C. levels of evidence 3 and 4 (lowest).  'Expert consensus' in the absence of relevant studies. | A method proposed by the Haute Autorité de Santé (HAS) was used (HAS, 2010). This is based on critical review of the literature and the views of a multidisciplinary group of professionals. | Y | Y | Y | Y | N | N | Y |
| Staal et al., 2017 | Physiotherapy and Manual therapy | 3-5 years, update planned for 2018 at the latest | Evidence level: A1 (systematic review); A2 (RCT of good quality); B (RCT of poorer quality or other comparative study, e.g. matched cohort, non-RCT), C (non-comparative study); D (expert opinion)  Recommendation level: Level 1 (A1 or multiple A2 studies) - "*It has been demonstrated that*.."; Level 2 (multiple B studies) – “*It is possible that*.."; Level 3 (1 x A2 or B study) - "*There are indications that..*"; Level 4 (C studies or consensus only) - "*The working group is of the opinion that* .." | Not described | Y | Y | Y | Y | Y | N | N |
| Sundhedsstryrelsen, 2016a | Development/Working group consisting of 12 interdisciplinary clinicians; Reference group consisting of 11 interdisciplinary clinicians; Secretariat consisting of 4 persons from the Danish Health Authority | Every 3 years, or if new evidence emerges | Overall level of evidence (high, moderate, low, very low) and strength of recommendations (Strong, weak, good practice) determined using GRADE. | The guideline used the Danish Health Authorities recommendations that are based on the GRADE system | N | N | N | N | Y | Y | N |
| Sundhedsstryrelsen, 2016b | Development/Working group consisting of 11 interdisciplinary clinicians; Reference group consisting of 12 interdisciplinary clinicians; Secretariat consisting of 6 persons from the Danish Health Authority | Every 3 years, or if new evidence emerges | Overall level of evidence (high, moderate, low, very low) and strength of recommendations (Strong, weak, good practice) determined using GRADE. | The guideline used the Danish Health Authorities recommendations that are based on the GRADE system | N | N | N | N | Y | Y | N |
| van Wambeke et al., 2017 | Multidisciplinary including GP, psychologists, physiotherapists, orthopaedic surgeon, anaesthesiologist, neurosurgeons | Ideally reviewed 5 years after publication to determine if all or part should be updated. (may be earlier if important new evidence published) | Overall level of evidence determined using GRADE (high, moderate, low, very low).  Strength of recommendation based on GRADE (strong/weak). Wording in guideline to reflect strength: 'offer'/’do not offer’ - strong recommendation for/against; 'consider'/’do not routinely offer’ - weak recommendation for/against.  Nb: based on NICE guideline. | NICE evidence and recommendations checked, summarised and added, and discussed in GDG meetings.  Before each meeting, GDG members asked to agree or not with NICE recommendation and strength suggested by KCE team. KCE made an overview of the agreement scores and the comments formulated by the GDG members. This structured the GDG meeting and enabled discussion of each comment (including of GDG members not present). In cases of disagreement, the topic was proposed for re-discussed at the next GDG meeting. Minutes highlighted changes made to recommendations and the reasons and were sent to every GDG member after each meeting. Four consultation rounds which each contained roughly 15 recommendations to be scored and commented were needed.  Final list of NICE and Belgium recommendations checked during the final GDG meeting (24 Jan 2017). | Y | Y | Y | N | N^$^ | N^$^ | N^$^ |
| Neck and low back pain | | | | | | | | | | | |
| Kasssolik et al. 2017 | Experts from the Polish Society of Physiotherapy, Polish Society of Family Medicine and College of Family Physicians in Poland | Not reported | Not reported | Not reported | N | N | N | Y | N | N | N |

^$^ - following this guideline, a clinical pathway was developed by KCE that addressed planning of care, practitioner education, and organisation and policy.
