# Supplementary material for: Evidence‐based treatment recommendations for neck and low back pain across Europe: A systematic review of guidelines
Source: Eur J Pain. 2020 Nov 12;25(2):275–95. doi: 10.1002/ejp.1679 (PMC7839780; doi:10.1002/ejp.1679)
Supplement: Supplementary file 5 — Appendix S5 [file EJP-25-275-s005.docx]

Supporting Information Appendix S5: Neck pain recommendations in European practice guidelines

Direction and strength of recommendations for each guideline (for symbol definition see Supporting Information Appendix S2). Green columns represent guidelines rated as high quality. **Abbreviation:** SC – self-care, SST – Sundhedsstyrelsen

| Guideline ID | SST 2016c | | Bier 2016 | | Monticone 2013 | Kasssolik 2017 | | Pohl 2018 | | SST 2015 |
| --- | --- | --- | --- | --- | --- | --- | --- | --- | --- | --- |
| Country | **DK** | | **NL** | | **IT** | **PL** | | **DE** | | **DK** |
|  | Recent onset non-specific neck pain | | Neck pain (Grades I-IV; Profiles A-D)^a^ | | Neck pain | Cervical back pain syndrome | | Cervical radiculopathy | | Recent onset cervical radiculopathy |
| Guideline quality | **High** | | **Low** | | **Low** | **Low** | | **Low** | | **High** |
| Reassurance |  | |  | |  |  | |  | |  |
| Reassurance | **O+** | | **/** - I-III (A-D) | |  | **/*** - acute | |  | |  |
| Advice and Education |  | |  | |  |  | |  | |  |
| Advice and Education |  | | **/** - I-III (A-D) | | **X** | **/*** - acute | | **//** | | **O+** |
| Written information | **X** | |  | |  |  | |  | |  |
| Bed rest |  | |  | |  | **X* -** 1-2 days selected cases | |  | |  |
| Remain active |  | | **/** - I-III (A-D) | |  |  | |  | | **O+** |
| Encourage exercise |  | | **/** - I-III (A-D) | |  | **/*** | |  | | **O+** |
| Avoid movement/activity that provokes radiating pain or other symptoms in the arm |  | | **/** - III (D) | |  |  | |  | |  |
| Continue/return to work |  | | **/** - I-III (A-D) | |  |  | |  | |  |
| Work-related/occupational advice |  | | **/** - I-III (A-D) | |  |  | |  | |  |
| Psychosocial aspects that delay/inhibit recovery |  | | **/** - I/II (C) | |  |  | |  | |  |
| Medication |  | |  | |  |  | |  | |  |
| Analgesics incl. for neuropathic pain |  | |  | |  | **/* -**  incl. SC | | **//** - offer early | |  |
| Paracetamol |  | |  | | **/ -** short-term |  | |  | | **O+** |
| NSAIDs | **O+ -** short term | |  | | **/ -** short-term | **/*** - SC | |  | | **O+ -** short term |
| Opioids including tramadol | **O+ -** short term | |  | |  |  | |  | | **O+ -** short term |
| Steroids |  | |  | | **/ -** short-term |  | |  | |  |
| Topical medications incl. NSAIDs | **/** | |  | |  | **/*** - SC | |  | |  |
| Injection/infiltration |  | |  | |  |  | |  | |  |
| Spinal epidural steroid injection (transforaminal route with imaging) |  | |  | |  |  | | **/** - severe persistent symptoms | |  |
| Thermotherapy |  | |  | |  |  | |  | |  |
| Thermotherapy incl. hot/ cold compress, bath, sauna |  | | **X***- I-III (B-D) | |  | **/*** | |  | |  |
| Thermotherapy + other treatment |  | | **O+** - I-III (B-D) - short-term | |  |  | |  | |  |
| Manual therapies |  | |  | |  |  | |  | |  |
| Manual therapy including mobilisation, manipulation and soft-tissue techniques | **X** | |  | | **//** - acute | **/*** | | **//** | | Massage: **O-;** Mobilisations: **/** |
| Manual therapy + other treatment | **/** | | Massage: **O+ -**I-III (B-D), short-term; Mobilisation + exercise: **O+ -** III (D), Thoracic mobilisation + exercise: **/ -** III (D) | | Manipulation/mobilisation + exercise: **//** - chronic; Massage with exercise + manipulation/mobilization: **/** - chronic |  | |  | |  |
| Exercise |  | |  | |  |  | |  | |  |
| Exercise programs/therapy |  | | **/** - I/III (B-D) | | **//** - chronic | **/*** | | **//** | | Neuro-muscular exercises: **/;** McKenzie Method exercises**: O+** |
| Exercise therapy + other treatment | **/** | | **//** - I/III (B-D) | |  |  | |  | |  |
| Postural therapies |  | |  | |  |  | |  | |  |
| Postural re-education |  | |  | |  | **/*** | |  | |  |
| Traction |  | |  | |  |  | |  | |  |
| Traction |  | | **O+** - III (D);  **X*** - I/II (B-C) | | **X** |  | |  | | **/** |
| Electrotherapies |  | |  | |  |  | |  | |  |
| Electrotherapy incl. light, laser, Low Level Laser Therapy (LLLT), pulsed electromagnetic, TENS, Ultrasound (US), shockwave, Magnetic, electromagnetic |  | | **X* -** I-III (B-D) | | LLLT: **/** - acute and chronic - short term; Pulsed electro-magnetic therapy: **O+** - acute and chronic, short term; TENS: **X** - acute and chronic | TENS (SC), Light, US, magnetic field, electro-magnetic: **/***; Intensive physical therapy e.g. high energy laser, shock-wave, electro-stimulation: **X*** - chronic | | **XX** | |  |
| Electrotherapies + other treatment |  | |  | | **/** - chronic |  | |  | |  |
| Orthotics |  | |  | |  |  | |  | |  |
| Cervical orthoses (soft and stiff collars) |  | | **O+** - III (D), short-term**;**  **X*** - I-II (B-C) | | **O-** | **/*** | | **O-** - intermittent use, severe pain, max 10-25 days | |  |
| Ergonomic |  | |  | |  |  | |  | |  |
| Cervical cushion |  | | **O+** - I-III (B-D) | |  |  | |  | |  |
| Taping/strapping |  | |  | |  |  | |  | |  |
| Kinesiology tape |  | | **O+ -** I-III (B-D) trauma-related, short-term | |  |  | |  | |  |
| Acupuncture |  | |  | |  |  | |  | |  |
| Acupuncture/dry needling | **/** | | **X* -** I-III (B-D) | | **// -** sub-acute and chronic, short term |  | |  | | **O-** |
| Psychological interventions |  | |  | |  |  | |  | |  |
| Psychological therapies incl. behavioural and cognitive-behavioural therapies |  | | **O+ -** I-III (B-D) | | **/** - chronic | **/*** - chronic | |  | |  |
| Multidisciplinary treatment/programs | |  | |  | | |  | |  | |
| Multidisciplinary treatment |  | |  | | **/** - chronic |  | | **/** - chronic w/o indication for surgery | |  |
| Work-related interventions | |  | |  | | |  | |  | |
| Workplace interventions |  | | **O+** - I-III (B-D), if work-related | |  |  | |  | |  |
| Imaging |  | |  | |  |  | |  | |  |
| Imaging |  | |  | |  | **X*** | | **//** - to confirm diagnosis | |  |
| Referral |  | |  | |  |  | |  | |  |
| To GP and/or occupational health officer |  | | **O+** - I-III (B-D), after 6 weeks if treatment not effective | |  |  | |  | |  |
| To GP or to the referring specialist |  | | **O+** - IV | |  |  | |  | |  |
| To physical therapist specialized in worker rehabilitation |  | | **O+** - I-III (A-D), where absenteeism or production loss without absenteeism | |  |  | |  | |  |
| To occupational health officer or a physical therapist specialised in worker rehabilitation |  | | **O+** - I-III (B-D) - where work-related factors suspected to impede recovery | |  |  | |  | |  |
| To occupational health and safety service (Arbodienst) |  | | **O+** - I-III (B-D) - in cases of absenteeism | |  |  | |  | |  |
| Encourage patient to contact GP, psychologist and/or psychosomatic therapist |  | | **O+** - I/II (C) - where psychosocial factors hinder recovery | |  |  | |  | |  |
| To surgeon/surgery |  | |  | |  |  | | **/** - chronic, refractory (appropriate method for anatomical findings)**; O+** - severe neurological symptoms | |  |
| Miscellaneous |  | |  | |  |  | |  | |  |
| Bioptron lamps |  | |  | |  | **/*** - SC | |  | |  |
| Ledotherapy lamps |  | |  | |  | **/*** - SC | |  | |  |
| Infra-red lamps |  | |  | |  | **/*** - SC | |  | |  |
| Bath salts with mud extracts, special water-pearling inserts or ozone |  | |  | |  | **/*** - SC | |  | |  |
| Magnetic mattress |  | |  | |  | **/*** - SC | |  | |  |

^a^ **Profile A**, neck pain grade I/II, normal course; **Profile B**, neck pain grade I/II, delayed course without dominant psychosocial influence; **Profile C**,
