# Supplementary material for: Evidence‐based treatment recommendations for neck and low back pain across Europe: A systematic review of guidelines
Source: Eur J Pain. 2020 Nov 12;25(2):275–95. doi: 10.1002/ejp.1679 (PMC7839780; doi:10.1002/ejp.1679)
Supplement: Supplementary file 6 — Appendix S6 [file EJP-25-275-s006.docx]

Supporting Information Appendix S6: Low back pain recommendations in European practice guidelines

Direction and strength of recommendations for each guideline (for symbol definition see Supporting Information Appendix S2). Green columns represent guidelines rated as high quality. **Abbreviation:** SC – self-care, SST - Sundhedsstyrelsen

| Guideline ID | Bons, 2017 | | SFMT, 2013 | | BAK, 2017 | | | Staal, 2017 | | | SST, 2016a | van Wambeke, 2017 | | | NICE, 2016 | | Kassolik, 2017 | | | Regione Toscana, 2015 | | Glocker, 2018 | | Schaafstra, 2015 | | | SST, 2016b |
| --- | --- | --- | --- | --- | --- | --- | --- | --- | --- | --- | --- | --- | --- | --- | --- | --- | --- | --- | --- | --- | --- | --- | --- | --- | --- | --- | --- |
| Country | **NL** | | **FR** | | **DE** | | | **NL** | | | **DK** | **BE** | | | **UK** | | **PL** | | | **IT** | | **DE** | | **NL** | | | **DK** |
|  | NSLBP | | Lumbar spine, in context of workers exposed to lifting and handling | | NSLBP +/- radiating, w/o radiculopathy | | | LBP, including specific and non-specific (profile 1-3)^a^ | | | recent onset NSLBP +/- leg pain | LBP and radicular pain | | | LBP and sciatica | | Lumbar-sacral back pain syndrome (LBP) | | | LBP +/- radiculopathy (sciatica or cruralgia)^$^ | | lumbar radiculopathy | | Lumbo-sacral radicular syndrome | | | recent onset lumbar radiculopathy |
| Guideline quality | **Low** | | **Low** | | **High** | | | **Low** | | | **High** | **High** | | | **High** | | **Low** | | | **Low** | | **Low** | | **Low** | | | **High** |
| Reassurance |  | |  | |  | | |  | | |  |  | | |  | |  | | |  | |  | |  | | |  |
| Reassurance |  | |  | |  | | | **/*** - Profiles 1-3 | | |  | **O+** | | |  | | **/*** - acute | | | **//** - general LBP & acute NSLBP | |  | |  | | |  |
| Advice and Education |  | |  | |  | | |  | | |  |  | | |  | |  | | |  | |  | |  | | |  |
| Advice and Education (including individualised) | **/*** | | **O+** - acute and subacute LBP; **O** - cLBP | | **//** | | | **/*** - Profiles 1-3 | | | **/** | **O+** | | | **O+** | | **/*** - acute | | | **//** - general LBP | |  | | **/*** - for a period of 6-8 weeks | | |  |
| Bed rest |  | |  | | **XX** | | | **X*** - max 2 days if only way to control pain - Profiles 1-3 | | |  |  | | |  | | **X*** - acute, but in selected cases 1-2 days | | | **XX** - general LBP, acute NSLBP; **O-** - acute radiculopathy (a few days if severe sciatica) | | **X***- max 4 days | | **X*** - only for a few days if symptoms severe | | |  |
| Remain active | **/*** | |  | | **//** - subacute and chronic**; O+** acute LBP | | | **/*** - Profiles 1-3 | | | **/** | **O+** | | | **O+** | |  | | | **//** - general LBP & acute NSLBP; **O+** acute radiculopathy | | **/*** | |  | | | **/** |
| Encourage physical exercise (unsupervised) |  | | **//** | |  | | | **/*** - Profiles 1-3 | | |  | **O+** | | | **O+** | | **/*** | | | **//** - general LBP | |  | | **/*** - for a period of 6-8 weeks | | |  |
| Continue/return to work |  | |  | |  | | | **/*** - Profiles 1-3 | | |  |  | | |  | |  | | | **//** - general LBP & acute NSLBP; **O+** - acute radiculopathy | |  | |  | | |  |
| Medication |  | |  | |  | | |  | | |  |  | | |  | |  | | |  | |  | |  | | |  |
| Analgesics (general) |  | |  | |  | | |  | | |  |  | | |  | | **/* -** incl. SC | | |  | |  | |  | | |  |
| Paracetamol | **/***^b^ | |  | | **X** | | |  | | | **X** | **X** - as single medication | | | **X** * - as single medication | |  | | | **//** - acute nsLPB; **O+ -** acute radiculopathy; **O** - cLBP | | **/*** ^b^ | | **/*** ^b^ | | |  |
| NSAIDs | **/***^b^ | |  | | **/** - traditional NSAIDs**; O+** *-* COX2i | | |  | | | **X** | **/** | | | **/** | | **/*** - SC | | | **// -** acute nsLPB; **O+** - acute radiculopathy**; O** – cLBP | | **/*** ^b^ | | **/*** ^b^ | | |  |
| Opioids (including tramadol) +/- paracetamol (or NSAIDs) | **/***^b^ – weak opioids for severe acute LBP; weak or strong opioids for cLBP with severe limitation.  Short-term | |  | | **//** - weak opioids if other analgesics not effective;  **X** – transdermal | | |  | | | **X** | **O+** - acute LBP +/-radicular pain if NSAID contraindicated, not tolerated or ineffective; **X -** cLBP | | | **/**- acute LBP only if an NSAID is contraindicated, not tolerated or ineffective; **X*** - cLBP | |  | | | **//** - acute NSLBP; **O+** - acute radiculopathy; **O** - cLBP | | **/*** ^b^ | | **/*** ^b^ | | |  |
| Steroids |  | |  | |  | | |  | | |  |  | | |  | |  | | | **XX** - acute NSLBP; **O+** - acute radiculopathy, short term; **O** – chronic pain | |  | |  | | |  |
| Antidepressants including SSRIs, SNRIs, Tricyclics | **X*** | |  | | **X** - in general; **O+** if depressive comorbid symptoms | | |  | | |  | **XX** - SSRIs; **X** - Tricyclics & SNRIs | | | **X*** - SNRIs, SSRIs, Tricyclics | |  | | | **O** - cLBP | | **X*** - SSRIs; **/***- Tricyclics. chronic pain | |  | | |  |
| Anticonvulsants/Antiepileptics including gabapentin, pregablin, carbamazepine, topiramat | **X*** | |  | | **X** | | |  | | |  | **XX** - LBP +/- radicular pain in absence of neuropathic pain | | | **X*** | |  | | | **XX** - acute NSLBP; **O-** - acute radiculopathy; **O** - cLBP | |  | |  | | |  |
| Muscle relaxants including diazepines/benzodiazepines | **X*** | |  | | **X** - acute, **XX** - chronic NSLBP. BUT **O+** - acute and chronic if non-drug measures or non-opioid analgesics not effective | | |  | | |  | **XX** - (skeletal MRs) | | |  | |  | | | **//** - (centrally acting MRs)  acute NSLPB. 2^nd^ line only; **O** - cLBP | |  | | **X*** | | |  |
| Antibiotics |  | |  | |  | | |  | | |  | **XX** | | |  | |  | | |  | |  | |  | | |  |
| Metamizol |  | |  | | **O+** - short term if NSAIDs not effective | | |  | | |  |  | | |  | |  | | |  | |  | |  | | |  |
| Flurpirtin |  | |  | | **XX** | | |  | | |  |  | | |  | |  | | |  | |  | |  | | |  |
| Uridine monophosphate (UMP) |  | |  | | **XX** | | |  | | |  |  | | |  | |  | | |  | |  | |  | | |  |
| Topical medications/NSAIDS | **/*** - NSAIDs | |  | | **XX** - NSAIDs | | |  | | |  |  | | |  | | **/*** - SC | | |  | |  | |  | | |  |
| Injection/infiltration |  | |  | |  | | |  | | |  |  | | |  | |  | | |  | |  | |  | | |  |
| Spinal injections [for non-specific LBP] | **X*** | |  | |  | | |  | | |  | **XX** | | | **X*** | |  | | | **O** - cLBP | | **O** | | **X*** | | |  |
| Spinal epidural steroid injection |  | |  | |  | | |  | | |  | **/** - [sub-]acute (at least 2-3 weeks) and severe radicular pain | | | **/ -** acute, severe sciatica; **X*** - neurogenic claudication in central spinal canal stenosis | |  | | | **O+** - acute radiculopathy; **O** - cLBP | |  | | **/*** - if symptoms severe, > 6-8 weeks' duration, and surgery not indicated | | | **X** |
| Other injections including intravenous, intramuscular, infiltration of trigger points and ligaments, intradiscal infiltration, prolotherapy, Botulium toxin |  | |  | | **XX** | | |  | | |  |  | | |  | |  | | | **O** - cLBP | |  | |  | | |  |
| Thermotherapy |  | |  | |  | | |  | | |  |  | | |  | |  | | |  | |  | |  | | |  |
| Thermotherapy including local heat, hot/cold compresses, baths, sauna |  | |  | | heat: **O+;** cold: **X** | | | **/* -** Profile 2, if impaired joint function | | |  |  | | |  | | heat/cold: **/*** | | | Local heat: **//** - acute NSLBP; **O** - cLBP | | heat: **O** | |  | | |  |
| Manual therapies |  | |  | |  | | |  | | |  |  | | |  | |  | | |  | |  | |  | | |  |
| Manual therapy including mobilisation, manipulation and soft-tissue techniques |  | |  | | **XX** - acute (massage); **O** - manipulation/mobilisation | | | **/* -** Profile 2, if impaired joint functionality;  **X*** - Profiles 1&3 | | | **/** |  | | |  | | **/*** | | | **Massage: XX** - acute NSLBP  **O-** - acute radiculopathy; **O** - cLBP. **Manipulation**: **//** - acute NSLBP (after 2-3 weeks and before 6 from onset); **O** - cLBP | | **X*** | | **X*** | | | **/** |
| Manual therapy in combination with other treatment |  | |  | | **O+** - subacute/ chronic | | |  | | |  | **/** | | | **/** | |  | | |  | | **Massage + exercise:** **/*** - - subacute & chronic; **Massage + electro-therapies:** **/*** - acute radiculopathy with limited mobility | |  | | |  |
| Exercise |  | |  | |  | | |  | | |  |  | | |  | |  | | |  | |  | |  | | |  |
| Exercise programs/therapy | **/*** - especially for those who have been unable to stay active | |  | | **// -** subacute/ chronic; **O** - acute | | | **/*** - Profiles 2 & 3 | | | **/** | **/** | | |  | | **/*** | | | **XX -** specific exercises - acute NSLBP | |  | | **/*** | | | **/** |
| Group exercise programmes/back schools |  | |  | | **/ -** subacute and chronic**; O+** - chronic recurrent | | |  | | |  |  | | | **/** | |  | | |  | | **/*** - chronic only | |  | | |  |
| Postural therapies |  | |  | |  | | |  | | |  |  | | |  | |  | | |  | |  | |  | | |  |
| Postural therapies e.g. Alexander therapy, postural re-education |  | |  | |  | | |  | | |  | **O** | | | **O** | | **/*** - SC | | |  | |  | |  | | |  |
| Traction |  | |  | |  | | |  | | |  |  | | |  | |  | | |  | |  | |  | | |  |
| Traction |  | |  | | **XX** | | | **O- -** Profiles 1-3 | | |  | **XX** | | | **X*** | | **/*** | | | **O -** cLBP | |  | |  | | |  |
| Taping/strapping |  | |  | |  | | |  | | |  |  | | |  | |  | | |  | |  | |  | | |  |
| Kinesiotaping |  | |  | | **XX** | | |  | | |  |  | | |  | |  | | |  | |  | |  | | |  |
| Electrotherapies |  | |  | |  | | |  | | |  |  | | |  | |  | | |  | |  | |  | | |  |
| Electrotherapy including laser therapies, TENS, PENS, shortwave diathermy, ultrasound (US), ultrashortwave, inferential, magnetic field, electromagnetic, light therapy, shockwave, electrostimulation |  | |  | | **XX** | | | **O- -** Profiles 1-3 | | |  | **XX** | | | **X*** | | TENS (incl SC), US, (electro)-magnetic field, light therapy - **/*; I**ntensive physical therapy (e.g. shockwave, high energy laser or electro-stimulation) - **X*** | | | **XX** - acute NSLBP; **O-** - acute radiculopathy; **O** - cLBP | |  | |  | | |  |
| Orthotics |  | |  | |  | | |  | | |  |  | | |  | |  | | |  | |  | |  | | |  |
| Orthoses including belts, corsets, foot orthotics, insoles, rocker shoes, pull-ups, walking stick, elbow crutches and bands |  | | **X** | | **XX** | | |  | | |  | **XX** | | | **X*** | | **/*** | | | **XX** - acute NSLBP; **O-** - acute radiculopathy; **O** - cLBP | |  | |  | | |  |
| Complementary and alternative therapies (CAM) | | | |  | | |  | | |  | | |  | | |  | | |  | | | |  | | |  | |
| Acupuncture |  | |  | | **O+** - acute (if other treatments not effective, short term) and chronic | | |  | | | **X** | **O** | | | **X*** | |  | | |  | |  | |  | | | **O-** |
| CAM (acupuncture and TCM, phytotherapy, homeopathy, manual therapies) |  | |  | |  | | |  | | |  |  | | |  | |  | | | **O+** | |  | |  | | |  |
| Phytotherapeutics |  | |  | | **O+; X** – Harpago-phytum procumbens | | |  | | |  |  | | |  | |  | | |  | |  | |  | | |  |
| Topical phytotherapeutics |  | |  | | **X** - *Symphytum officinale*; **O+** - Capsaicin | | |  | | |  |  | | |  | |  | | |  | |  | |  | | |  |
| Psychological interventions |  | |  | |  | | |  | | |  |  | | |  | |  | | |  | |  | |  | | |  |
| Psychological therapies including behavioural and cognitive behavioural therapies | **/*** - after 12 weeks, especially if struggling to manage | |  | | **//** - subacute LBP depending on psycho-social risk profile | | |  | | |  |  | | |  | | **/*** | | |  | | **/*** - chronic pain | |  | | |  |
| Psychological therapies + other treatment (exercise) |  | |  | |  | | |  | | |  | **/** | | | **/** | |  | | |  | |  | |  | | |  |
| Progressive muscle relaxation |  | |  | | **O-** - acute LBP, **O+ -** subacute LBP or those with stress, anxiety etc, - may prevent chronic pain in this group; **/ *-*** chronic LBP | | |  | | |  |  | | |  | |  | | |  | |  | |  | | |  |
| Multidisciplinary treatment/program | |  | | | |  | | |  | | | | |  | | | |  | | |  | | | |  | | |
| Multidisciplinary treatment including multidisciplinary biopsychosocial rehabilitation (MBR) programs | **/*** - cLBP and motivated to receive further treatment | | **/** - subacute or cLBP, **O -** acute LBP | | **//** - cLBP | | | **/*** - profiles 2 & 3 with delayed recovery | | |  | **/** - persistent LBP or radicular pain: with psychosocial obstacles to recovery, or when previous evidence-based management has not been effective | | | **/ -** persistent LBP or sciatica with significant psychosocial obstacles to recovery or when previous treatments have not been effective. | |  | | | **O+** - subacute & chronic NSLBP: disability is high, or recent onset and patient strongly motivated to resolve | |  | |  | | |  |
| Work-related interventions |  | |  | |  | | |  | | |  |  | | |  | |  | | |  | |  | |  | | |  |
| Work-based interventions including rehabilitation programmes (exercises, preferably with workplace visits, workplace adjustments, or other interventions on the part of the employer) |  | | **// -** employees; **/** - for employers (context of 1^o^ or 2^o^ prevention) | | **/** | | |  | | |  |  | | |  | |  | | | **//** - acute NSLBP | |  | |  | | |  |
| Return to work programmes |  | |  | | **//** | | |  | | |  | **O+** | | | **O+** | |  | | |  | |  | |  | | |  |
| Collaborate with company doctor, company physical therapist or occupation health and safety service |  | |  | |  | | | **/* -** Profile 2 - if sick leave >4 weeks; **/*** - Profile 3 - if recovery impeded by heavy physical work, prolonged sick leave, a labour dispute, or if collaboration expected to promote the recovery | | |  |  | | |  | |  | | |  | |  | |  | | |  |
| Imaging |  | |  | |  | | |  | | |  |  | | |  | |  | | |  | |  | |  | | |  |
| Imaging | **X*** - unless  suspicion of serious pathology^†^ | | **XX** - except if red flags^†^; Repeat imaging not recommended within a year, unless there is a new clinical indication for it | | **XX** - acute or persistent LBP if no red flags^†^, OR repeat imaging (w/o change in clinical presentation); **//** - severe and disabling LBP not improved after 6 weeks treatment OR Referral for further investigations, including appropriate imaging if red flags^†^ | | |  | | | MRI: **X** | **X -** unless red flags^†^, or if its expected result may lead to change in management | | |  | | **X*** | | |  | | **X*** - unless red flags^†^ | | **X*** - unless red flags/  suspicion of serious pathology^†^ | | | MRI: **X** |
| Referral |  | |  | |  | | |  | | |  |  | | |  | |  | | |  | |  | |  | | |  |
| To family doctor |  | |  | |  | | | **/*** **-** Profile 3, if treatment has had no effect after 3-6 weeks | | |  |  | | |  | |  | | |  | |  | |  | | |  |
| To manual therapist |  | |  | |  | | | **/* -** Profile 2, if impaired joint functionality | | |  |  | | |  | |  | | |  | |  | |  | | |  |
| To family doctor, company doctor and/or psychologist |  | |  | |  | | | **/* -** Profile 3, if serious or persistent psychosocial factors hamper recovery | | |  |  | | |  | |  | | |  | |  | |  | | |  |
| For specialist assessment (neurophysiopathological evaluation) |  | |  | |  | | |  | | |  |  | | |  | |  | | | **O+** - acute radiculopathy, if persistent pain, or atypical lower limb responses, or new or progressive motor deficits | |  | |  | | |  |
| To surgeon/surgery (also included here where guideline addresses a particular surgical intervention and gives context e.g. failure of non-surgical intervention) | **X*** | |  | | **XX** | | |  | | |  | **//** - only after failure of a non-surgical evidence-based multimodal management, and after evaluation in a multi-disciplinary consultation | | | **/** - for failed non-surgical treatment, and source of pain from structures supplied by the medial branch nerve and moderate or severe LBP at the time of referral OR for sciatica with failed non-surgical treatment with sciatica consistent radiological findings | |  | | | **O+** **-** acute radiculopathy, only after 1 month of conservative therapy if severe and disabling, with no improve-ment or worsening or clinical evidence of compressed nerve root; <1 month if neurological worsening, severe pain and resistant to conservative therapy or appearance of red flags; **O** – cLBP | | **/*-** specific indications only [cauda equina symptoms, severe neurological signs, or in case of persistent problems and clear nerve compression and no response to conservative treatment] | | **/*** - after 12 weeks, if persistent (severe) symptoms | | | **O+ -** within 12 weeks in cases where severe and disabling pain persists despite non-surgical treatment |
| Miscellaneous |  | |  | |  | | |  | | |  |  | | |  | |  | | |  | |  | |  | | |  |
| Spa treatments |  | |  | |  | | |  | | |  |  | | |  | |  | | | **O -** cLBP | |  | |  | | |  |
| Ozone therapy |  | |  | |  | | |  | | |  |  | | |  | |  | | | **O -** cLBP | |  | |  | | |  |
| Medullary stimulations |  | |  | |  | | |  | | |  |  | | |  | |  | | | **O -** cLBP | |  | |  | | |  |
| ‘Taking it slowly/easy’ |  | |  | |  | | |  | | |  |  | | |  | |  | | | **O -** cLBP | |  | |  | | |  |
| Bioptron lamps |  | |  | |  | | |  | | |  |  | | |  | | **/*** - SC | | |  | |  | |  | | |  |
| Ledotherapy lamps |  | |  | |  | | |  | | |  |  | | |  | | **/*** - SC | | |  | |  | |  | | |  |
| Infra-red lamps |  | |  | |  | | |  | | |  |  | | |  | | **/*** - SC | | |  | |  | |  | | |  |
| Bath salts with mud extracts, water-pearling inserts or ozone |  | |  | |  | | |  | | |  |  | | |  | | **/*** - SC | | |  | |  | |  | | |  |
| Magnetic mattress |  | |  | |  | | |  | | |  |  | | |  | | **/*** - SC | | |  | |  | |  | | |  |
| Shock-absorbing or anti-fatigue flooring |  | | **X** | |  | | |  | | |  |  | | |  | |  | | |  | |  | |  | | |  |

^$^ recommendation strength given en masse per type of back pain (For and against), not on individual intervention basis

† further details regarding red flags given in Supporting Information Appendix S7

^a^ **Profile 1:** normal course; **Profile 2 / 3:** abnormal course, without / with dominant presence of psychosocial factors impeding recovery respectively

^b^ stepwise according to guidance: paracetamol, NSAIDs, opiate
