# Supplementary material for: Evidence‐based treatment recommendations for neck and low back pain across Europe: A systematic review of guidelines
Source: Eur J Pain. 2020 Nov 12;25(2):275–95. doi: 10.1002/ejp.1679 (PMC7839780; doi:10.1002/ejp.1679)
Supplement: Supplementary file 7 — Appendix S7 [file EJP-25-275-s007.docx]

Supporting Information Appendix S7: Red flags in the context of imaging in low back pain recommendations

| Guideline ID, publication date | Condition | Imaging recommendations | Red flags/serious pathologies |
| --- | --- | --- | --- |
| BÄK, *et al.,* 2017 | Nonspecific low back pain with or without radiating pain | Referral for further investigations, including appropriate imaging if there are red flags / warning signs. | (1) **fracture/osteoporosis**: severe trauma, minor trauma in older people or those wit (likely) osteoporosis; treatment with systemic steroids; (2) **infection**: general malaise, previous bacterial infection, immune suppression, recent back surgery, severe pain at night; (3) radiculopathy: severe progressive symptoms, **cauda equina symptoms** (sensory deficits, incontinence, paresis); (4) **tumour/metastases**: older age, cancer history, unexplained weight loss, loss of appetite, severe night pain; (5) **axial spondylitis**: morning stiffness, peripheral arthritis, etc |
| Bons *et al.,* 2017 | Non-specific low back pain | Imaging in case of suspected serious pathology. | (1) suspicion of **rupture aneurysm of aorta**: age >40, acute severe pain, history of aorta aneurysm, symptoms or signs of threatened circulation (fainting, dizziness, etc); (2) symptoms of **cauda equina**: sensory deficits; severe radicular pain; incontinence; recent back surgery; (3**) unexplained progressive course; fever and night sweats; cancer history** |
| Glocker *et al.*, 2018 | Lumbar radiculopathy | CT or MRI only in case of red flags. | (1) previous trauma in older people with increased risk of fragility **fracture**, including those with osteoporosis; (2) suspicion of **tumour** or **infection**: fever, unexplained weight loss, increasing pain at night; (3) **cauda equina syndrome:** progressive paresis, incontinence |
| Schaafstra *et al.* 2015 | Lumbo-sacral radicular syndrome | Imaging when there is suspicion of serious pathology. | Red flags/signs and symptoms: (1) **severe pain, cancer history, recent back surgery, unexplained weight loss, fever, possible Lyme's disease**; (2) **cauda equina symptoms**: incontinence, severe sensory deficits or loss of strength; (3) when surgery may be indicated . |
| Société Française de Médecine du Travail (SFMT) (2013) | Lumbar spine, in context of workers exposed to lifting and handling | Standard X-ray generally not recommended in patients 20 to 55 years old, except if there are suspect clinical signs /red flags.  Second-line imaging (CT, MRI, scintigraphy, PET) not recommended in the absence of suspicious clinical signs / red flags. | (1) **Progressively worsening non-mechanical pain**, present at rest and in particular during night; (2) **widespread neurological symptoms** (deficit in the control of the bladder or anal sphincters, motor impairment of the legs, cauda equina syndrome); (3) **paraesthesia in the pubis or perineum**; (4) **Significant trauma** e.g. fall from a height; (5) **Unexplained weight loss**; (6) **History of cancer**, **presence of febrile syndrome**; (7) **IV drug use, or prolonged use of corticosteroids**; (8) **Significant structural deformation of the spinal column**; (9) **Chest pain (= back pain)**; (10) **Age of onset <20 years or >55 years**; (11) **Fever**; (12) **Altered of general condition**. |
| van Wambeke *et al.* 2017 | LBP and radicular pain | Do not routinely offer imaging in the absence of red flags.  Only refer for imaging if expected results may lead to change in management (e.g. if an invasive intervention is being considered). | (1) **Neurological emergencies:** widespread neurological symptoms; progressive neurological symptoms; cauda equina symptoms: saddle anaesthesia/hypoesthesia, urinary retention, urinary or faecal incontinence, isolated sexual disfunction; (2) **Fracture (traumatic)**: severe low back pain following significant/high-energy trauma; back pain following trauma with ankylosing spondylitis; (3) Vascular signs indicative of **torn aortic aneurysm** alongside low back pain or sock; (4) **Fracture (pathological):** history/risk of osteoporosis, long-term corticosteroid use, thoracic pain, older age, unexplained weight loss, cancer history; (5) **infection:** objective signs, IV drug use, patient immunocompromised, unexplained weight loss, previous/concurrent systematic infection or risk of infection, recent surgical intervention, urinary or cutaneous infection. |
